# The C-terminal 18 Amino Acid Region of Dengue Virus NS5 Regulates its Subcellular Localization and Contains a Conserved Arginine Residue Essential for Infectious Virus Production

**DOI:** 10.1371/journal.ppat.1005886

**Published:** 2016-09-13

**Authors:** Moon Y. F. Tay, Kate Smith, Ivan H. W. Ng, Kitti W. K. Chan, Yongqian Zhao, Eng Eong Ooi, Julien Lescar, Dahai Luo, David A. Jans, Jade K. Forwood, Subhash G. Vasudevan

**Affiliations:** 1 Program in Emerging Infectious Diseases, Duke-NUS Medical School, Singapore; 2 School of Biomedical Sciences, Charles Sturt University, Wagga Wagga, New South Wales, Australia; 3 Department of Microbiology and Immunology, Yong Loo Lin School of Medicine, National University of Singapore, Singapore; 4 School of Biological Sciences, Nanyang Technological University, Singapore; 5 UPMC UMRS CR7—CNRS ERL 8255-INSERM U1135 Centre d’Immunologie et des Maladies Infectieuses. Centre Hospitalier Universitaire Pitié-Salpêtrière, Faculté de Médecine Pierre et Marie Curie, Paris, France; 6 Lee Kong Chian School of Medicine, Nanyang Technological University, Singapore; 7 Nuclear Signalling Laboratory, Department of Biochemistry and Molecular Biology, Monash University, Melbourne, Victoria, Australia; Institut Pasteur, FRANCE

## Abstract

Dengue virus NS5 is the most highly conserved amongst the viral non-structural proteins and is responsible for capping, methylation and replication of the flavivirus RNA genome. Interactions of NS5 with host proteins also modulate host immune responses. Although replication occurs in the cytoplasm, an unusual characteristic of DENV2 NS5 is that it localizes to the nucleus during infection with no clear role in replication or pathogenesis. We examined NS5 of DENV1 and 2, which exhibit the most prominent difference in nuclear localization, employing a combination of functional and structural analyses. Extensive gene swapping between DENV1 and 2 NS5 identified that the C-terminal 18 residues (Cter_18_) alone was sufficient to direct the protein to the cytoplasm or nucleus, respectively. The low micromolar binding affinity between NS5 Cter_18_ and the nuclear import receptor importin-alpha (Impα), allowed their molecular complex to be purified, crystallised and visualized at 2.2 Å resolution using x-ray crystallography. Structure-guided mutational analysis of this region in GFP-NS5 clones of DENV1 or 2 and in a DENV2 infectious clone reveal residues important for NS5 subcellular localization. Notably, the *trans* conformation adopted by Pro-884 allows proper presentation for binding Impα and mutating this proline to Thr, as present in DENV1 NS5, results in mislocalizaion of NS5 to the cytoplasm without compromising virus fitness. In contrast, a single mutation to alanine at NS5 position R888, a residue conserved in all flaviviruses, resulted in a completely non-viable virus, and the R888K mutation led to a severely attenuated phentoype, even though NS5 was located in the nucleus. R888 forms a hydrogen bond with Y838 that is also conserved in all flaviviruses. Our data suggests an evolutionarily conserved function for NS5 Cter_18_, possibly in RNA interactions that are critical for replication, that is independent of its role in subcellular localization.

## Author Summary

DENV NS5 is critical for virus RNA replication and an important drug target based on its high sequence conservation across serotypes, and the successful development of potent drugs that target the homologous NS5B of hepatitis C virus. NS5 also mediates other functions that are important for innate and adaptive immune responses by the infected host. Extensive gene swapping and functional analyses between NS5 of DENV serotypes 1 and 2, that are the two most disparate in terms of nuclear vs cytoplasmic localization of NS5 identified the last 18 amino acid residues of the ~900 amino-acid residues long protein to be responsible for subcellular localization. Because this region is very flexible and not easily seen in crystal structures of DENV NS5, co-crystals of the newly discovered peptide region with importin α were obtained. Structure-based mutations introduced into a DENV2 infectious clone showed that the proline to threonine at position 884 resulted in NS5 being mostly cytoplasmic without affecting virus replication. However mutation of arginine 888, which is conserved in all flaviviruses, to alanine resulted in a completely non-viable virus, suggesting that the C-terminal region is essential for NS5 function irrespective of its role in subcellular location.

## Introduction

Dengue is one of the most prevalent and important mosquito-borne viral disease and endemic in more than 100 tropical and subtropical countries. Nearly half of the world’s population is thought to be at risk of dengue infection and ~100 million symptomatic dengue cases are reported each year [1, 2]. Dengue infection in humans, caused by any one of the four distinct, but closely related Dengue virus serotypes (DENV1-4), is mostly asymptomatic but those who manifest the disease exhibit a wide-spectrum of clinical symptoms, ranging from self-limiting dengue fever (DF) to life-threatening severe dengue characterized by vascular leakage, thrombocytopenia and bleeding [3]. The current treatment for dengue infection is mainly supportive. A tetravalent vaccine that is partially effective has been licensed in Brazil, Mexico and the Philippines. There are no antiviral drugs that can be used to prevent or treat dengue infection. Classical vector control strategies remain the cornerstone in preventing transmission, and molecular vector control approaches are gaining prominence [4].

The DENV single-stranded positive-sense RNA genome (~11kb) contains a single open reading frame that is flanked by 5’- and 3’-untranslated regions. The open reading frame is translated into an ~3,300 amino acid residue polyprotein precursor that is cleaved by host and viral proteases into three structural (C, prM and E) and seven nonstructural (NS; NS1, NS2A, NS2B, NS3, NS4A, NS4B and NS5) proteins [5]. Among the NS proteins, NS5 is the largest (103 kDa, 900 amino acids) and the most conserved with a sequence identity of around 70% among the four serotypes [5, 6]. It contains methyltransferase (MTase) [7–9] and putative guanylyltransferase activities [10] at its N-terminal region, and the C-terminal region carries out RNA-dependent RNA polymerase (RdRp) activity [11–13]. In infected cells, NS5 works in concert with NS3 to participate in type 1 cap formation [14] and viral RNA replication [15–18]. In addition to its enzymatic activities, DENV NS5 has been shown to modulate the host immune response and to induce the expression and secretion of DHF-associated immunomediators, IL-6 and IL-8 [19, 20]. More specifically, the N-terminal region of DENV2 NS5 can bind to human STAT2 [21], and promote UBR4-mediated STAT2 degradation that results in the inhibition of type I interferon signalling [21–24].

NS3 N570 and NS5 K330 are key residues involved in the interaction between NS3 and NS5 that act cooperatively to replicate the viral RNA within discrete replication complexes (RCs) [15, 25] but the mechanistic details such as the order in which the various processes occur during the early stages in viral RNA replication remains unknown. The introduction of K330A mutation into a DENV2 infectious clone does not yield any viable virus while the N570A mutation, although severely attenuated (5% of WT level), is able to accumulate negative-strand RNA at the early stages of viral RNA replication. However, because of the impaired interaction with NS5, its positive-strand RNA synthesis function is muted [17, 26]. Even though NS5 from the four serotypes of DENV are highly similar in structure and function it has been shown to be transported into the nucleus utilising the host nuclear transport machinery composed of importin-alpha and beta-1 to varying degrees [27, 28]. The phenotypic differences in subcellular localization can be ascribed to adaptive changes acquired during virus evolution but its essentiality and role in pathogenesis remains contentious. The importin-alpha recognition of basic-residue-rich nuclear localization signal (NLS) on NS5 facilitates its passage across the nuclear pore complex [27–29]. Previous NS5 localization studies have suggested that the a/bNLS (residues 369–389) [29, 30] and bNLS (residues 320–368) [31] reside within an interdomain linker region spanning residues 320–405 until the atomic resolution structure of DENV RdRp and recent functional studies [32] revealed that these residues map within the palm and thumb subdomains raising the possibility that they are not accessible for interaction with the importin machinery of the host cell.

Using an in-house generated NS5 antibody [33], DENV1-4 NS5 was shown to differentially localize in infected cells. In particular, DENV1 NS5 is predominantly cytoplasmic while DENV2, 3 and 4 NS5 appear in the nucleus of infected cells [28]. In order to explore the causes underlying these distinct phenotypes from a structural and functional perspective, we hypothesized that a sequence within DENV1 NS5 plays a dominant role in influencing the function of a/bNLS. We performed extensive gene swapping between DENV1 and 2 full-length NS5 proteins to search for the signal sequence. We unexpectedly discovered a C-terminal monopartite NLS in DENV2 NS5 that resides within residues 883–900. This region of NS5 is very flexible and was not observed in our previous crystal structures of DENV NS5 [11, 13, 34] or JEV NS5 [35] but appears to play a role in NS5 oligomerization in a more recently published structure [36]. In this work, structural, biochemical, computational and reverse genetics data provide a compelling picture that the C-terminal region of DENV NS5 alone is sufficient to determine the localization differences between DENV serotypes. Our data also suggest that the C-terminal region may carry out an evolutionarily conserved function that is essential for flavivirus replication and pathogenesis. The implications of this discovery on DENV pathogenesis are discussed.

## Results

### The C-terminal 18 residues (Cter_18_) of DENV NS5 confer its nucleocytoplasmic shuttling ability

**Fig 1:**
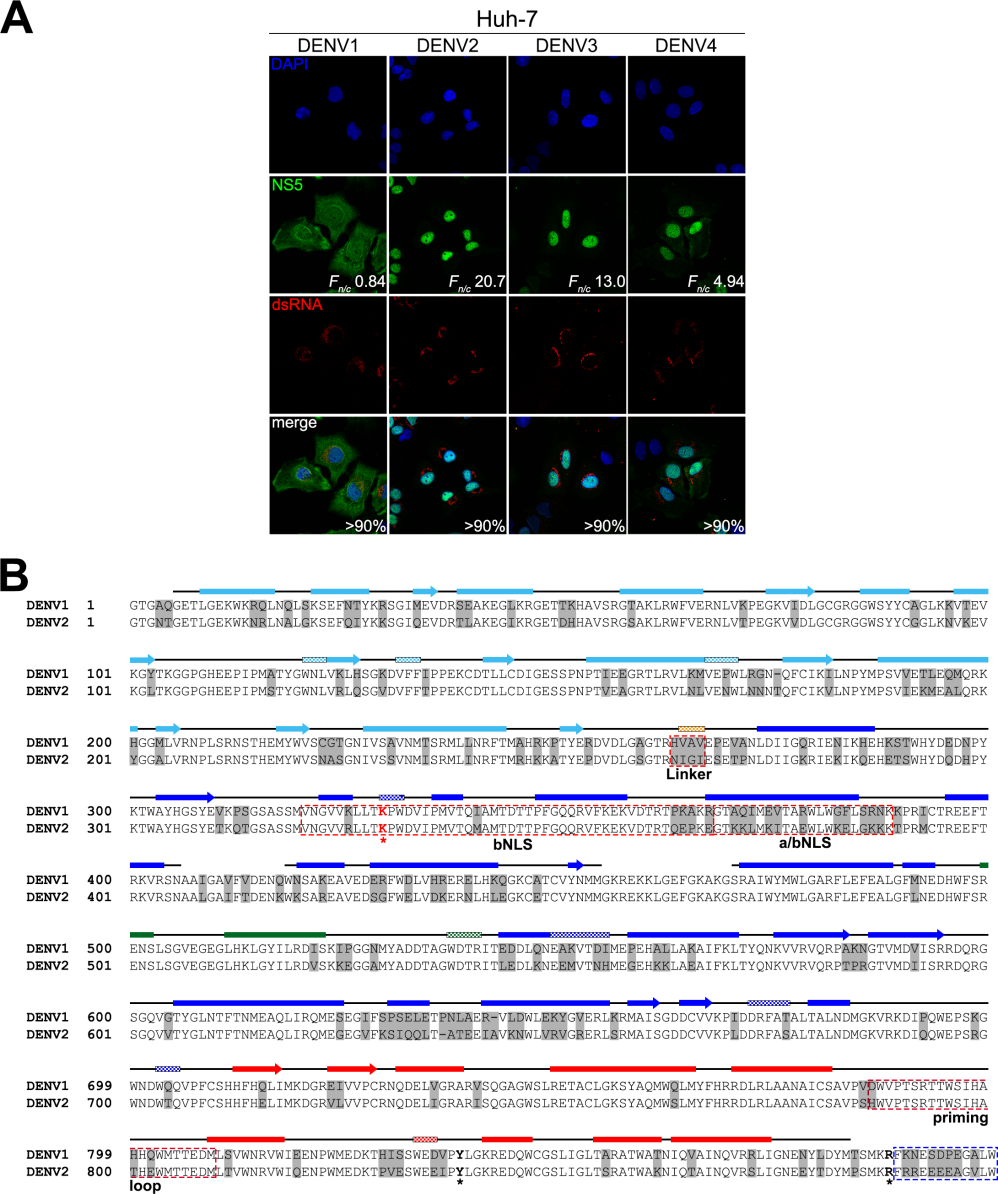
Subcellular localization of NS5 in DENV1-4 infected cells and sequence alignment of DENV1 and 2 NS5. (**A**) Huh-7 cells were infected with DENV1-4 at MOI 10 and the infected cells (>90%) were analysed for presence of NS5 (green) and dsRNA (red) by IFA at 24h post-infection. Digitized images were captured by Zeiss LSM 710 upright confocal microscope by 63× oil immersion lens. Image analysis was performed on digitized images of NS5 staining with ImageJ software [52] to determine nuclear to cytoplasmic fluorescence ratio (*F*_*n/c*_) as done previously [17, 29, 30, 42]. (**B**) Sequence and structural analysis of DENV1 NS5 aligned against DENV2 NS5. The alignment was performed using Clustal Omega and the numbering of the alignment is based on DENV2 NS5. The PDB file of DENV3 NS5 protein (PDB: 4V0Q, [34] was used as the input file for secondary structure depiction. The secondary structures are indicated by solid boxes (α-helices), checked boxes (3_10_ helix) and arrows (β-sheets) above the sequence alignment and they are coloured light blue for the Mtase domain, orange for linker (residues 264–273) and blue for the fingers, green for the palm and red for the thumb of the RdRp domain. The bNLS sequence (residues 320–368) [31], the a/bNLS sequence (residues 369–389) [29, 30] and the priming loop sequence (residues 786–809) [13] are boxed in red. Within bNLS, K330 (red asterisk and bold) is important for NS3–NS5 interaction [26]. NS5 residues that are not identical to DENV1 and DENV2 are highlighted in grey. Other highlighted residues Y838 and R888 are discussed in the text. The electron density of the last 12 amino acid residues (boxed in blue) are generally missing in most DENV NS5 crystal structures (PDB: 2J7U, 4C11 and 4V0Q). The GenBank accession numbers of DENV1 and DENV2 are EU081230 and EU081177, respectively.

**Fig 2:**
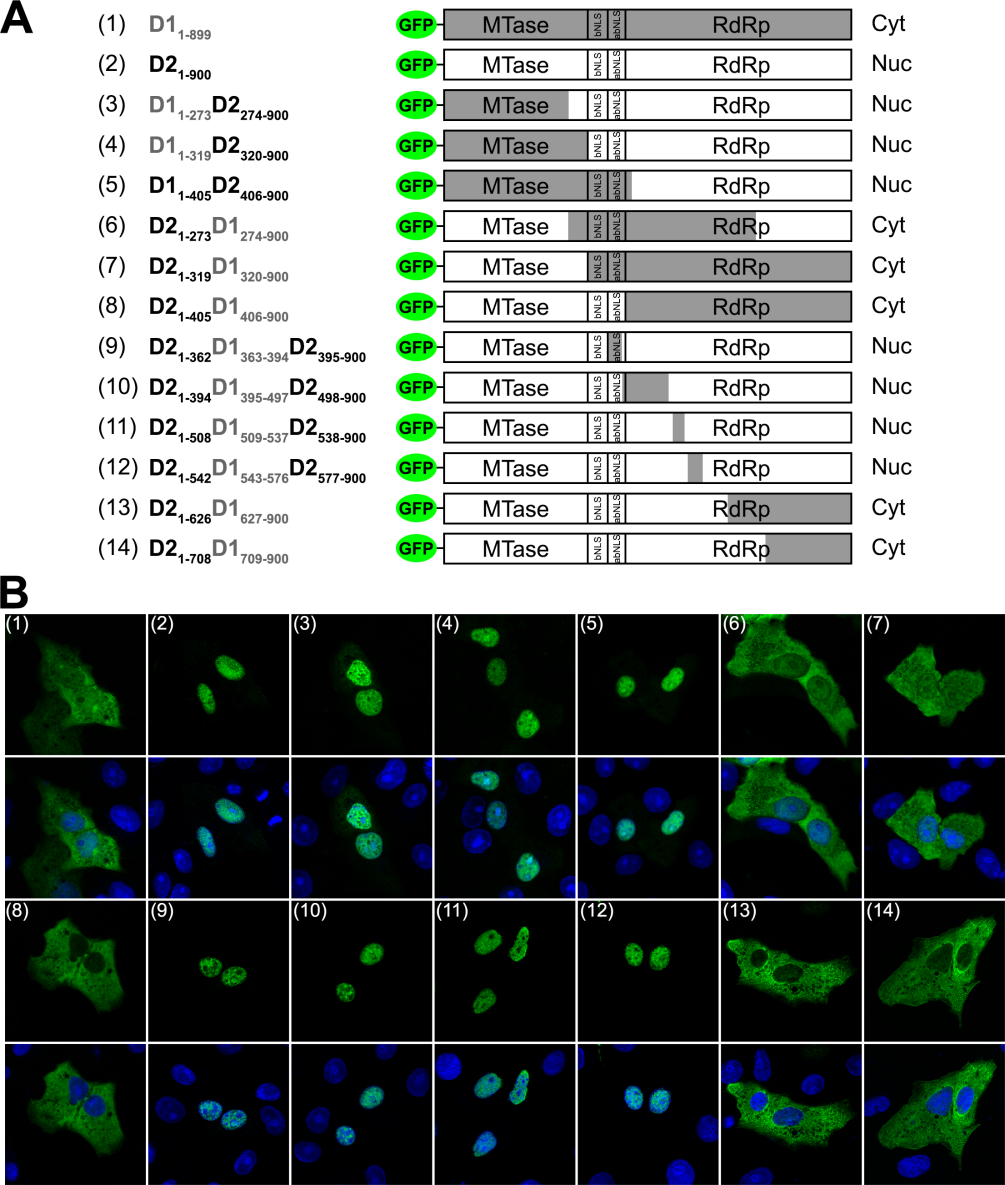
Subcellular localization of chimeric DENV1 and 2 NS5 fused to GFP. (**A**) Schematic diagrams of WT DENV1 and 2, and chimeric DENV1/2 GFP-NS5 protein mammalian expression constructs. The indicated NS5 gene regions of DENV1 and DENV2 were exchanged by gene swapping and their localization were analysed in Vero cells (B). The construct number and gene boundaries that were defined for swapping are indicated on the left and the exchanged segments are shaded in the schematic (DENV1 in grey and DENV2 in white). The observed subcellular localization of these constructs are indicated on the right (Cyt for predominately cytoplasmic localization and Nuc for predominately nuclear localization). (**B**) The GFP-NS5 protein constructs described in A were transfected into Vero cells and fixed at 24-h post-transfection. Anti-GFP (ab6556 IgG, 1:1000) antibody was used for immunostaining and digitized images were captured by Zeiss LSM 710 upright confocal microscope by 40× oil immersion lens. The construct numbers used in Fig 2A are indicated in parenthesis in the images. The image used are representative of two independent experiments.

To assess the NS5 subcellular localization of DENV1-4, infected Huh-7 cells were examined and quantified using confocal laser scanning microscopy (CLSM) image analysis (Fig 1A, >90% infectivity). We found DENV1 NS5 distributed somewhat equally between the nucleus and cytoplasm with an *F*_*n/c*_ of around 0.84 ± 0.22 (n = 57) while DENV2, 3 and 4 NS5 are localized to the nucleus (DENV2 *F*_*n/c*_ = 20.75 ± 1.05, n = 47; DENV3 *F*_*n/c*_ = 13.01 ± 0.63, n = 51; and DENV4 *F*_*n/c*_ = 4.94 ± 0.32, n = 49). Similar observations were also seen in DENV1-4 infected A549 and Vero cells (S1A and S1B Fig, respectively). To explore the importance of nuclear NS5 and the implications of the serotype-dependent differences in the localization pattern, we initially focused on NS5 from DENV1 and 2 as examples of NS5 proteins that did not or did localize to the nucleus and exhibited the greatest difference in *F*_*n/c*_. Firstly, we carried out extensive domain shuffling in an ectopically expressed full-length GFP-NS5 protein in order to better understand the functional elements within NS5 that contributed to differential subcellular localization. The gene segments encoding the functional domains and motifs, namely the MTase and RdRp domains (residues 1–273 and 274–900 respectively, Fig 1B), the newly defined short interdomain linker region (residues 263–272), and the residues 320–405 which are thought to be a hot spot for protein interactions including bNLS (residues 320–368) [31] and a/bNLS (residues 369–389) [29, 30] were swapped by overlap PCR reactions to generate the corresponding GFP-NS5 plasmids (Fig 2A). Transfected Vero cells that expressed the various GFP constructs as intact proteins were fixed at 24 h post-transfection and stained with anti-GFP for CLSM (Fig 2B). The GFP-NS5 fusion constructs that contained residues 406–900 of DENV2 NS5, namely D1_1-273_D2_274-900_, D1_1-319_D2_320-900_ and D1_1-405_D2_406-900_ (corresponding to construct 3, 4 and 5, respectively, in Fig 2A) were predominantly localized in the nucleus (Fig 2B). In contrast, the chimeric NS5 proteins that contained residues 406–900 of DENV1 NS5, namely D2_1-273_D1_274-900_, D2_1-319_D1_320-900_ and D2_1-405_D1_406-900_ (corresponding to construct 6, 7 and 8, respectively, in Fig 2A) were predominantly localized in the cytoplasm (Fig 2B). Taken together, these results suggest that the region responsible for the differential subcellular localization of DENV1 and 2 NS5 is located within the C-terminal end of RdRp domain, from residues 406–900.

In order to further delineate the sequence responsible for differential subcellular localization within residues 406–900, we focused on exchanging gene segments that satisfy both requirements (i) presence of sequence variation and (ii) absence of structural motif (PDB: 4V0Q [34] and 4K6M [35])(Fig 1B). As shown in Fig 2A and 2B, chimeric GFP-NS5 proteins of DENV2 that contained residues 363–394, 395–497, 509–537, 543–576 of DENV1 (corresponding to construct 9, 10, 11 and 12 respectively, in Fig 2A) were found predominantly in the nucleus. In contrast, the chimeric DENV2 NS5 proteins that contained residues 627–900 and 709–900 of DENV1 (corresponding to construct 13 and 14, respectively, in Fig 2A) were found in the cytoplasm. This series of gene segment shuffling delineated residues 709–900 of DENV2 NS5 as the sequence element responsible for the observed differential subcellular localization (Fig 2B, construct 13). Even though D2_1-708_D1_709-900_ (Fig 2B, construct 13) contained all the previously characterized signal sequences (ie. bNLS and a/bNLS) required for nuclear transport, its localization was predominantly cytoplasmic compared to D2_1-900_ (Fig 2B, construct 2). Close inspection of the C-terminal 190 amino acid residues of DENV1 and 2 NS5 revealed a relatively lower sequence conservation (67% sequence similarity), particularly in the last 18 amino acid residues (Fig 1B). This region is also poorly conserved within DENV3 and DENV4 NS5 (S2 Fig). Furthermore, the last 12 amino acid residues are thought to be highly flexible and either not modelled in crystallographic structures [11, 13, 34], or only modelled in a subset of molecules within the asymmetric unit where crystal packing may induce a stable conformation [36].

**Fig 3:**
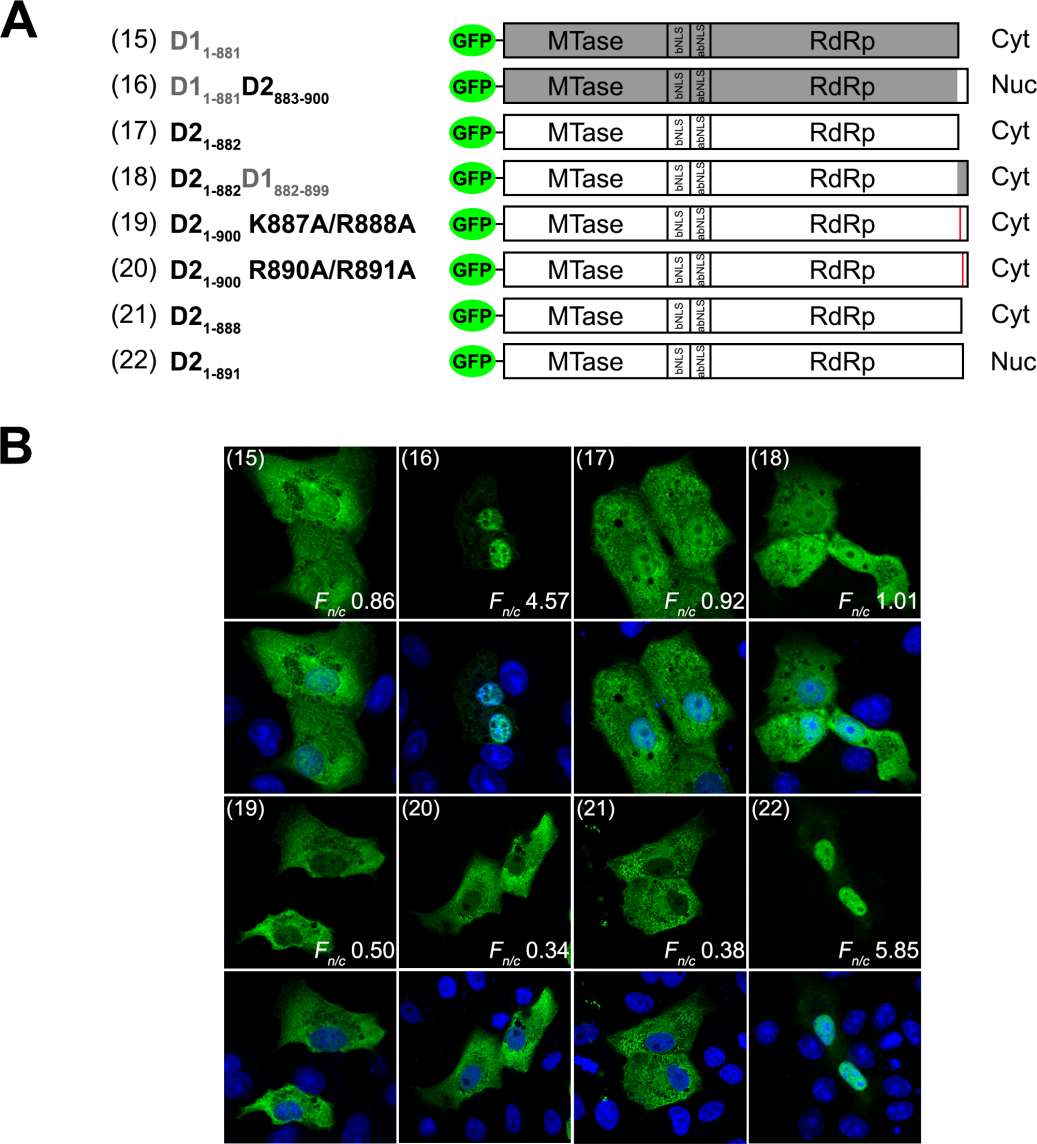
DENV1 and 2 NS5 C-terminal residues 883–900 confer differential sub-cellular localization. **(A)** Schematic diagram of full-length and truncated DENV1 and 2, and chimeric full-length DENV1/2 fused to GFP. The plasmid annotation is as described in Fig 2A. **(B)** The GFP-NS5 protein constructs described in A were transfected into Vero cells and fixed at 24-hour post-transfection. Anti-GFP (ab6556 IgG, 1:1000) antibody was used for immunostaining and digitized images were captured by Zeiss LSM 710 upright confocal microscope by 40× oil immersion lens. The construct numbers used in Fig 3A are indicated in parenthesis in the images. Nuclear to cytoplasmic fluorescence ratio (*F*_*n/c*_) as previously described [17, 29, 30, 42] are indicated and data are shown as mean *F*_*n/c*_, n ≥ 30 cells from a single assay, representative of two independent experiments.

To test the functional relevance of the C-terminal region, we truncated GFP-NS5 by introducing a stop codon at residue 882 or 883 of DENV1 and DENV2 NS5 respectively (Fig 3A, construct 15 and 17, respectively). Surprisingly, C-terminal truncated DENV2 NS5 protein, D2_1-882_ (construct 17) that contains the previously characterized conventional a/bNLS [29, 30] was mostly localized to the cytoplasm (Fig 3B). Both C-terminal truncated and full-length DENV1 NS5 proteins, D1_1-881_ and D1_1-900_ respectively (Fig 3A, construct 15 and Fig 2A, construct 1) were mainly cytoplasmic. Collectively, this data suggested that residues 883–900 contain the determinants for subcellular localization of NS5 from DENV1 and 2.

We tested the hypothesis that replacing residues 883–900 of DENV1 NS5 with the corresponding DENV2 NS5 sequence should drive the former, predominantly into the nucleus (Fig 3A, construct 16). The DENV1_1-882_DENV2_883-900_ chimeric NS5 localized predominantly in the nucleus (Fig 3B, construct 16). Replacing residues 883–900 of DENV2 NS5 with the corresponding DENV1 sequence (Fig 3A, construct 18) caused NS5 protein to be localized in the cytoplasm (Fig 3B, construct 18). To ensure that the phenomenon is not cell-type specific these constructs were also transfected into BHK-21 or HEK293T cells (S3A and S3B Fig, respectively), where similar observations were made. Taken together, our findings imply that C-terminal residues 883–900 of NS5 contain the sequence elements that account for the differential subcellular localization of DENV1 and 2 NS5.

### The Cter_18_ of DENV2 NS5 contains a monopartite nuclear localization signal

Due to the unexpected finding that residues 883–900 of DENV2 NS5 were sufficient for localization to the nucleus, we examined the sequence for the presence of a potential NLS (S2 Fig) and found that the basic residues at position 887–888 and 890–891 of DENV2 could possibly contribute to binding to nuclear import receptors for translocation to the nucleus [37]. To test the importance of these basic residues for nuclear accumulation of full-length NS5, we introduced alanine substitutions at these positions, as well as nonsense mutations immediately after these positions into DENV2 GFP-NS5. Alanine substitutions of K887/R888 and R890/R891 of DENV2 NS5 resulted in predominantly cytoplasmic localization (Fig 3A and 3B, constructs 19 and 20, respectively). Similarly, truncation immediately after the first basic cluster K887/R888 (known as D2_1-888_, Fig 3A and 3B, construct 21) also resulted in predominantly cytoplasmic localization. Remarkably, the ability for DENV2 NS5 to nuclear-localize was restored when a truncation was introduced immediately after the second basic cluster R890/R891 (known as D2_1-891_, Fig 3A and 3B, construct 22). The first basic cluster K887/R888 is also present in DENV1 (corresponding to residue 886 and 887) but the second cluster is K889/N890 compared to R890/R891 in DENV2. Given the subtle charge differences in the second cluster within the C-terminal region between the two proteins it was unclear why DENV1 NS5 was mostly cytoplasmic (to be discussed later). We decided to explore the structure and function of this region with respect to its binding to nuclear import proteins.

### The Cter_18_ of DENV1-4 NS5 interact differentially with importin-alpha

**Fig 4:**
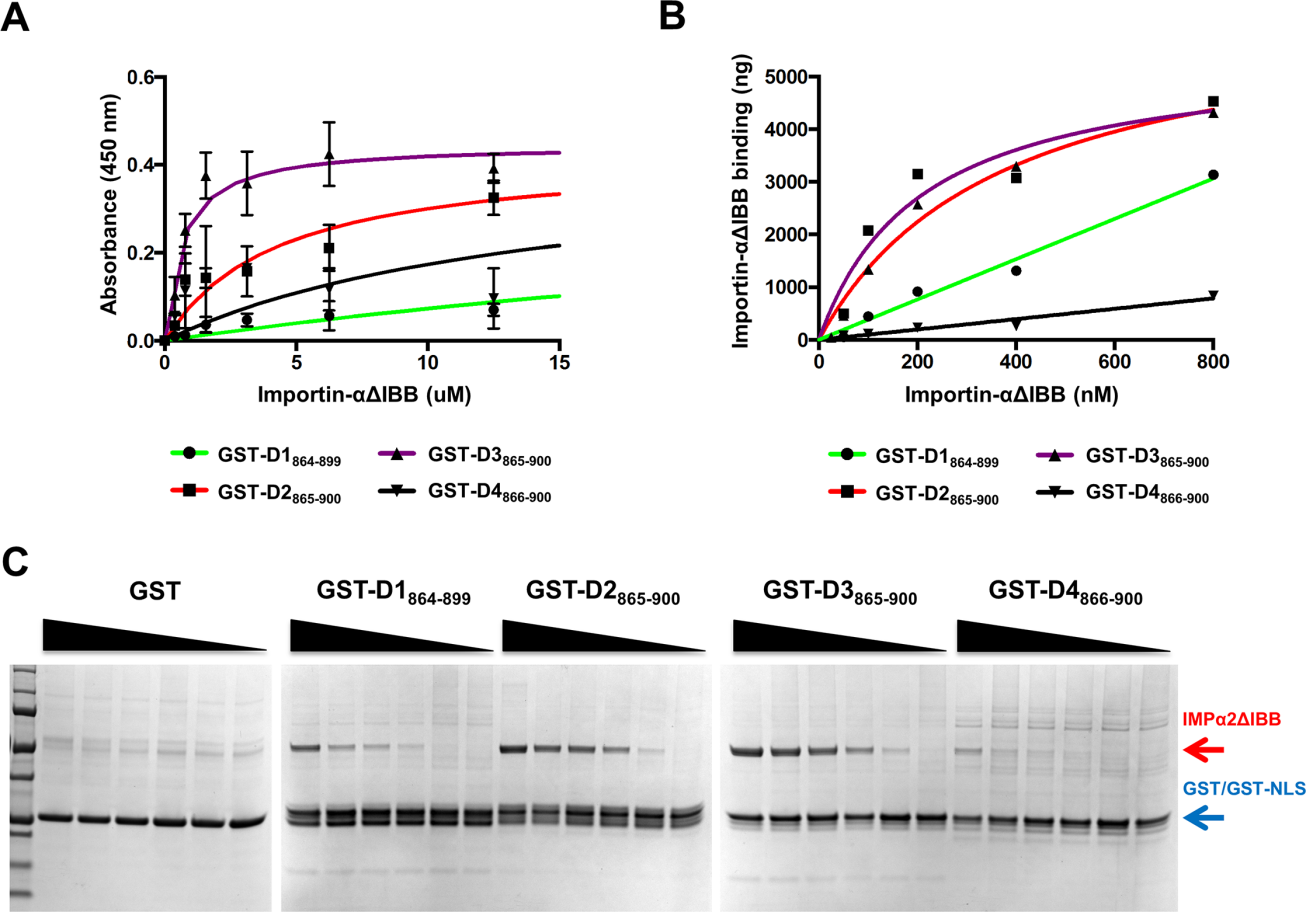
Characterization of the binding of DENV1-4 NS5 residues 865–900 to Impα. **(A)** After pre-incubation of glutathione coated plate with GST-NLS fusion protein, 2-fold serially diluted of Impα (starting from 12.5 μM) was added. Bound Impα protein was detected with anti-6x His tag HRP antibody. Following development with the colorimetric substrate TMB, the reaction was stopped and absorbance at 450nm was taken. Data were fitted to one site specific binding equation using Graphpad Prism 6.0f from triplicate measurements of two independent experiments.**(B and C)** Result of binding assay performed with glutathione beads that were incubated with GST-NLS fusion protein. **(B)** 2-fold serially diluted of Impα (starting from 800 nM) was incubated with GST-NLS fusion protein in a final volume of 50 ml. After incubation, beads were sedimented and protein was eluted using GST B buffer. **(C)** Elutled proteins were visualised on SDS-PAGE and analysis was performed using one site specific binding function in Graphpad Prism 6.0f. Results are shown as the mean ± SD of duplicates from two independent experiments.

Since our gene swapping studies were carried out for DENV1 and 2 NS5 we decided to explore the importance of the Cter_18_ across all four serotypes. We hypothesized that binding to the nuclear import receptor Impα will vary according to the observed subcellular localization (Fig 1A). To test this biochemically we compared the affinity of residues 865–900 of DENV1-4 NS5 (based on DENV2 NS5 numbering) for Impα lacking the auto-inhibitory importin beta binding domain (Impα, Fig 4A and 4B) [38]. A solid-phase binding assay was established, where residues 865–900 of DENV1-4 NS5 were recombinantly expressed as a C-terminal GST-fusion protein and captured by a glutathione microtitre plate. Serially diluted Impα protein was added to the plate, washed, and detected colorimetrically by probing with HRP-conjugated anti-His that binds to the His_6_ tag of Impα. As shown in Fig 4A, GST-DENV2_865-900_ and GST-DENV3_865-900_ (K_d_ = 4.53 ± 0.61 μM and 0.66 ± 0.16 μM, respectively) bound stronger to Impα than GST-DENV1_865-899_ and GST-DENV4_865-901_ (K_d_ = 53.96 ± 6.59 μM and 18.54 ± 5.13 μM, respectively).

To confirm this result in a different assay system, a GST pull down assay was performed using glutathione-agarose beads and serially diluted Impα, with visualisation and quantitation of bound Impα performed by SDS-PAGE and ImageJ respectively (Fig 4B and 4C). The results confirm that DENV2 and 3 C-terminal residues bind Impα, whilst the binding observed in the NS5 for DENV1 and DENV4 NLSs are very weak. Similar binding affinity values were obtained, with strong binding for GST-DENV2_865-900_ and GST-DENV3_865-900_ (K_d_ = 0.27 ± 0.11 μM and 0.37 ± 0.11 μM, respectively), whilst the binding of DENV1 and 4 were too weak to quantify. Overall, the affinity of DENV2 and 3 NS5 for importin by *in vitro* assay are similar while the affinity of DENV1 and 4 NS5 are weak. The differences in affinity measurement are within the expected range noted for well characterised targets such as SV40 T-ag NLS which can vary from 10–1000 nM depending on the methodolgy that is used [38, 39].

Together, our biochemical binding data appear to be consistent with the subcellular localization in infected cells for DENV1, 2 and 3 where DENV1 NS5 is mainly cytoplasmic whilst the protein from DENV2 and 3 are mainly localized in the nucleus. DENV4 NS5 deviated from the expected because it did not appear to bind Impα in our binding assays and yet the protein is mainly localized to the nucleus in the infected cells. Interestingly, this lack of binding may be explained by previous bioinformatic analysis which predicted that the C-terminal of DENV 4 NS5 is probably phosphorylated at Ser894 prior to nuclear localization in the infected cell [40] (see Discussion).

### Structure of Impα bound to Cter_18_ of DENV2 and 3 NS5

**Fig 5:**
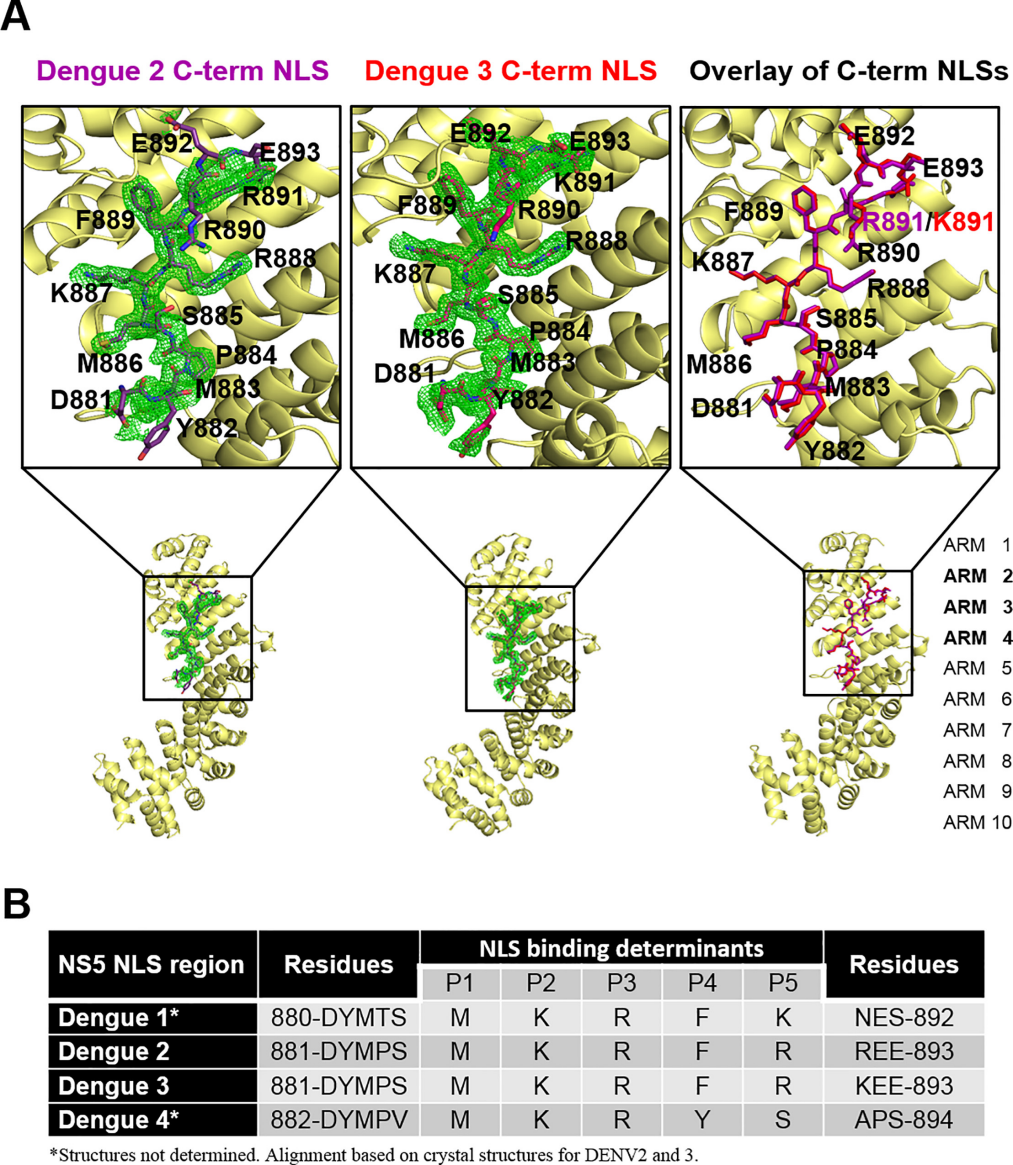
Structure of the DENV2 and DENV3 Cter_18_-Impα complex. **(A and B)** Atomic resolution structures of DENV2 Cter_18_-Impα complex and DENV3 Cter_18_-Impα complex. **(A)** DENV2 Cter_18_ (purple) and DENV3 Cter_18_ (red), in complex with Impα (yellow) reveal they are both monopartite NLSs and bind Impα in the major binding pocket (ARMs 2–4). **(B)** The 3σ simulated annealing Fo-Fc omit map, supports this model and position of the c-terminal NLS residues. The biggest energetic contribution to the interaction is from K887 which forms a salt bridge with Impα residue D192, R888 is also critical for hydrogen binding Impα residues N188 and N228, as is R890 that forms hydrogen bonds with Impα residues N146 and Q181. **(C)** NLS binding determinants in the DENV1-4 Cter_18._

In order to examine the precise residues that mediate the interaction with importin-α, x-ray crystallography was undertaken of protein complexes comprised of Impα bound to the DENV C-terminal residues 865–900. To obtain a 1:1 complex between the nuclear import receptor and the C-terminal region from respective DENV NS5 serotypes, the GST-tagged DENV C-terminal fusion proteins were first immobilized on glutathione affinity resin, washed, and Impα added to the column. Following further washing and elution from the column, the NS5 Cter_18_:Impα complex was released from GST by thrombin cleavage, and passed through a size exclusion column to remove excess NS5 C-terminal peptide, and then repurified over a glutathione column to remove uncleaved purified GST products (see Methods). As expected the poor binding affinity of DENV1 and DENV4 C-terminal residues prevented their respective capture of Impα, and therefore precluded structural analysis. In contrast, the C-terminal region of DENV2 and 3 formed a stable complex with Impα throughout purification, and yielded well diffracting crystals (S4 Fig). Crystals of Impα:DENV2 and 3 C-terminal region diffracted to 2.2 Å and 2.1 Å resolution, respectively. Both crystals belong to the P2_1_2_1_2_1_ space group, and the structures solved by molecular replacement using the structure of importin-α (PDB: 3UL1) as a search probe (Fig 5). The final R/Rfree were 0.177/0.216 and 0.183/0.207 for the Impα DENV2 and 3 C-terminal region respectively (see Table 1 for full data collection and refinement statistics). The nuclear import receptor Impα displayed the characteristic super-helical structure consisting of ten armadillo repeats (ARM), with each ARM repeat consisting of three α-helices. Strong positive residual electron density for residues 881–893 of DENV2 and 3 NS5 could be clearly discerned at the major NLS binding site of ARM2, 3, 4 of Impα, consistent with our cell localization data indicating that mutations within K887/R888 and R890/R891 can drastically impact nuclear accumulation.

**Table 1:** Data collection and refinement statistics. DENV2 NS5 C-terminal NLS: Impα and DENV3 NS5 C-terminal NLS: Impα data collection and refinement statistics:

	DENV2C-termNLS: Impα	DENV3C-termNLS: Impα
**Wavelength (**Å)	0.9537	0.9537
**Resolution range (**Å)	31.32–2.2 (2.279–2.2)	31.35–2.1 (2.175–2.1)
**Space group**	P 2_1_ 2_1_ 2_1_	P 2_1_ 2_1_ 2_1_
**Unit cell (**Å, °)	79.25 88.96 100.39 90 90 90	78.94 89.88 100.37 90 90 90
**Measured reflections**	160470 (11571)	172369 (14223)
**Unique reflections**	36643 (3080)	41892 (3446)
**Multiplicity**	4.4 (3.8)	4.1(4.1)
**Completeness (%)**	100 (99)	99 (100)
**Mean I/sigma (I)**	23.5 (7.6)	10.5 (1.9)
**Wilson B-factor (**Å^2^)	26.63	30.26
**R**_**pim**_	0.021(0.085)	0.053(0.455)
**Reflections used in refinement**	36589 (3555)	41840 (4172)
**Reflections used for R-free**	1825 (199)	2088 (175)
**R-work**	0.1770 (0.1943)	0.1825 (0.2659)
**R-free**	0.2158 (0.2678)	0.2066 (0.2900)
**Number of non-hydrogen atoms**	3684	3713
**macromolecules**	3373	3371
**Protein residues**	440	440
**RMS (bonds) (**Å)	0.006	0.010
**RMS (angles) (°)**	1.02	1.34
**Ramachandran favored (%)**	99	99
**Ramachandran allowed (%)**	1.4	1.4
**Ramachandran outliers (%)**	0	0
**Rotamer outliers (%)**	2.2	2.2
**Clashscore**	2.79	5.14
**Average B-factor (**Å^2^)	36.08	41.38
**macromolecules**	35.66	40.94
**solvent**	40.65	45.68

Statistics for the highest-resolution shell are shown in parentheses.

Extensive side chain and main chain interactions are established between DENV2 and 3 C-terminal NLS and Impα are summarized in Table 2. The NLSs of DENV2 and 3 NS5s are almost identical except for residue 891 where it is R and K respectively (Fig 5). Since this is the only variant residue close to the binding interface between the two structures and since this residue does not contact Impα, the analysis of both DENV2 and 3 NS5 C-terminal NLSs will be combined. One exception is that the side chain of Y882 from DENV2 NS5 hydrogen bonds with the side chain of E354 of Impα while in DENV3 NS5 it forms hydrogen bonds with Impα E354 and R315 (Table 2A). Main chain atoms of DENV2 NS5 P884, S885, M886 interact with side chain atoms of residues R238, W231, and N235 of Impα respectively (Fig 5A). The side chain of NS5 residue K887 (P2 position) makes a salt bridge with the carboxylic group of Impα residue D192 and hydrogen bonds with T155 and a backbone interaction is also established with G150. The DENV2 NS5 residue R888 side-chain (P3 position) forms extensive interactions with Impα; its guanidinium group forms hydrogen bond interactions with N228 and also its backbone interacts with Impα side-chain residues N188 and W184. The NS5 residue R890 (P5 position) form side chain interactions with Impα N181, and the backbone makes interactions with the side-chains of Impα residues W142 and N146. Additionally, E892 of NS5 form main chain interactions with S105. The NLS binding determinants, P1-P5, for DENV2 and 3 NS5 are listed in Fig 5B.

**Table 2:** PISA interface analysis of DENV C-terminal NLS Impα bond forming residues.

	C-terminal NLS	Distance [Å]	Impα	Bond*
**DENV2**	TYR 882 [OH]	3.55	GLU 354 [OE2]	H
	PRO 884 [O]	2.85	ARG 238 [NH1]	H
	SER 885 [O]	3.18	TRP 231 [NE1]	H
	MET 886 [O]	2.86	ASN 235 [ND2]	H
	ARG 888 [O]	2.87	TRP 184 [NE1]	H
	ARG 888 [O]	3.04	ASN 188 [ND2]	H
	ARG 890 [O]	3.04	TRP 142 [NE1]	H
	ARG 890 [O]	2.99	ASN 146 [ND2]	H
	LYS 887 [NZ]	3.10	GLY 150 [O]	H
	LYS 887 [NZ]	2.68	THR 155 [OG1]	H
	LYS 887 [NZ]	2.80	ASP 192 [OD1]	H
	ARG 888 [N]	2.73	ASN 188 [OD1]	H
	ARG 888 [NH1]	2.71	ASN 228 [OD1]	H
	ARG 888 [NH2]	2.88	ASN 228 [OD1]	H
	ARG 890 [N]	2.93	ASN 146 [OD1]	H
	ARG 890 [NH1]	2.63	GLN 181 [OE1]	H
	GLU 892 [N]	3.08	SER 105 [O]	H
	LYS 887 [NZ]	2.80	ASP 192 [OD1]	SB
**DENV3**	TYR 882 [OH]	3.84	ARG 315 [NH2]	H
	PRO 884 [O]	2.96	ARG 238 [NH1]	H
	SER 885 [O]	3.00	TRP 231 [NE1]	H
	MET 886 [O]	2.86	ASN 235 [ND2]	H
	ARG 888 [O]	2.95	ASN 188 [ND2]	H
	ARG 888 [O]	2.94	TRP 184 [NE1]	H
	ARG 890 [O]	3.03	TRP 142 [NE1]	H
	ARG 890 [O]	2.84	ASN 146 [ND2]	H
	TYR 882 [OH]	3.44	GLU 354 [OE2]	H
	LYS 887 [NZ]	2.79	ASP 192 [OD1]	H
	LYS 887 [NZ]	2.82	THR 155 [OG1]	H
	LYS 887 [NZ]	3.07	GLY 150 [O]	H
	ARG 888 [N]	2.72	ASN 188 [OD1]	H
	ARG 888 [NH1]	2.65	ASN 228 [OD1]	H
	ARG 888 [NH2]	2.80	ASN 228 [OD1]	H
	ARG 890 [N]	2.78	ASN 146 [OD1]	H
	ARG 890 [NH1]	2.63	GLN 181 [OE1]	H
	GLU 892 [N]	2.67	SER 105 [O]	H
	LYS 887 [NZ]	2.79	ASP 192 [OD1]	SB

* H = hydrogen bond, SB = salt bridge

**Fig 6:**
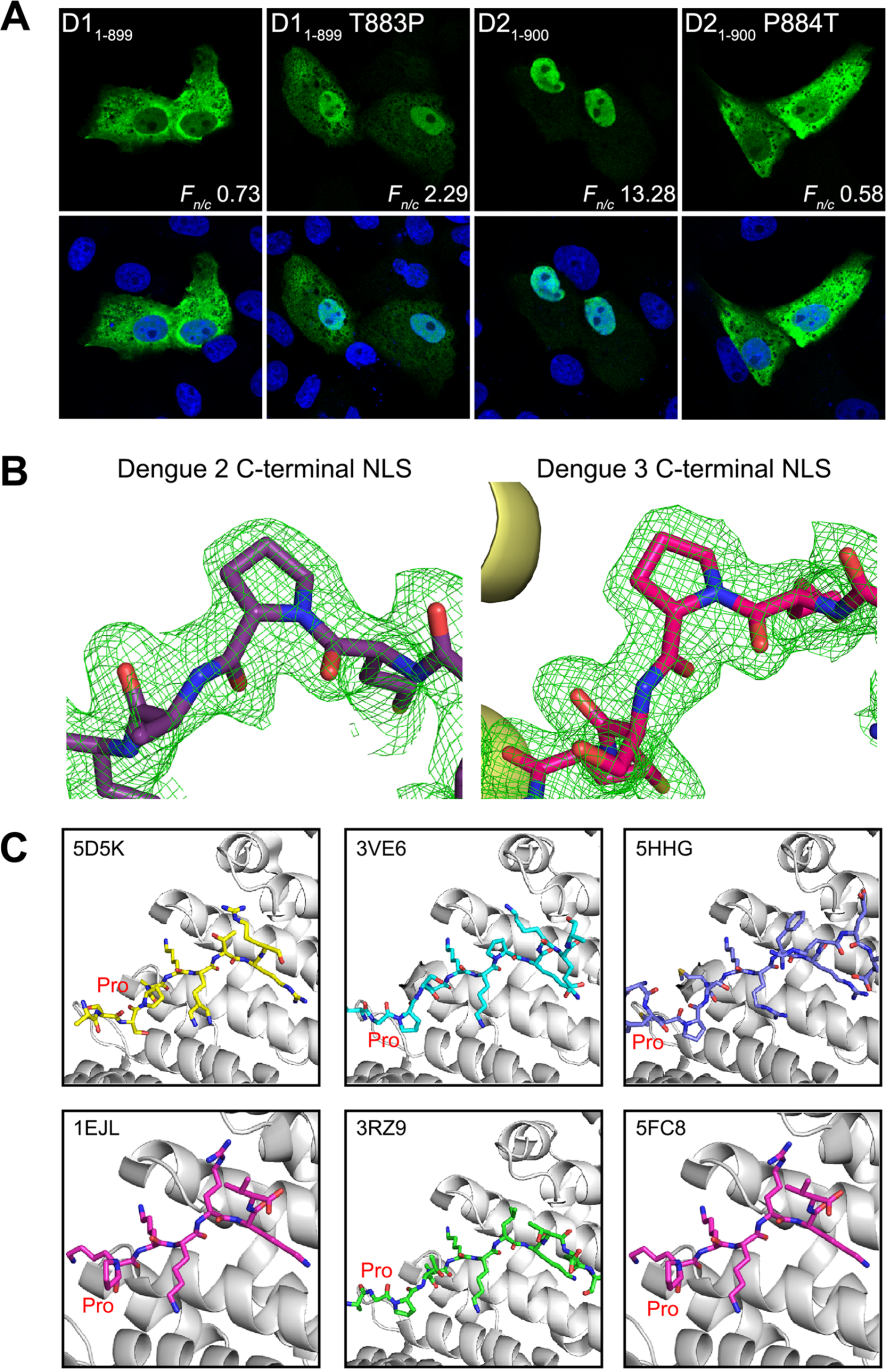
Impact of Cter_18_ conformation on DENV1 and DENV2 NS5 subcellular localization. **(A)** T883P and P884T mutations were introduced into DENV1 and 2 GFP-NS5 plasmids, respectively. The mutated plasmids were transfected into Vero cells and fixed at 24 hr post-transfection. Anti-GFP (ab6556 IgG, 1:1000) antibody was used for immunostaining and digitized images were captured by Zeiss LSM 710 upright confocal microscope by 63× oil immersion lens. Nuclear to cytoplasmic fluorescence ratio (*F*_*n/c*_) as previously described [17, 29, 30, 42] are indicated and data are shown as mean *F*_*n/c*_, n ≥ 30 cells from a single assay, representative of two independent experiments. **(B)** The Pro884 residue in both DENV2 and DENV3 NS5 C-terminal NLSs (shown in purple and pink respectively) is in the trans-conformation. The 3σsimulated annealing Fo-Fc omit map, supports this model and position of the Pro884 residue. Importin-alpha is shown as yellow ribbons. **(C)** Conservation of an N-terminal Pro residue in NLSs that bind the major binding site of importin-alpha. Importin-alpha is shown as grey ribbons and the Pro residues are labelled red. From left to right the top panel contains PARP2 (5D5K,shown in yellow); Venezuelan Encephalitis Virus (3VE6, shown in blue), DENV2 (5HHG; shown in purple); and the bottom panel contains SART3/TIP110 (5CTT; shown in magenta), Ku80 (3RZ9; shown in green), DENV3 (5FC8; shown in red).

The structures of the Impα complexes with DENV2 and 3 C-terminal peptides and models highlight key interacting residues required for their subcellular localization and as yet unknown functional modulation in viral life cycle. However, a sequence alignment of this C-terminal region of DENV1-4 NS5 reveals only minor differences in the NLS binding determinants across the four serotypes, including with DENV1 NS5 (Fig 5B). Based on the structures, we therefore hypothesized that residue P884 in the NS5 protein of DENV2 and 3 (and possibly DENV4 following specific phosphorylation) might be a key determinant for presenting the correct conformation of the C-terminal peptide region for binding. Conversely, DENV1 T883 (analogous position to P884) may not allow the peptide to adopt a conformation conducive to binding. To test this hypothesis, we introduced a T883P mutation for DENV1 GFP-NS5 and P884T for DENV2 GFP-NS5 (Fig 6A). Strikingly the T883P mutation in DENV1 NS5 resulted in a mostly nuclear localized protein while the P884T in DENV2 and 3 resulted in a mostly cytoplasmic protein, establishing that a “*trans*” conformation for the Pro residue (Fig 6B) is required for the C-terminal NLS peptide to adopt a correct conformation conducive to binding Impα. We note that a “*trans*” conformation of a proline residue preceding monopartite NLSs was also observed in other NLS-Impα structures deposited in the PDB (Fig 6C).

### Mutations in DENV2 C-terminal region in infectious cDNA affect virus replication and NS5 nuclear localization

**Fig 7:**
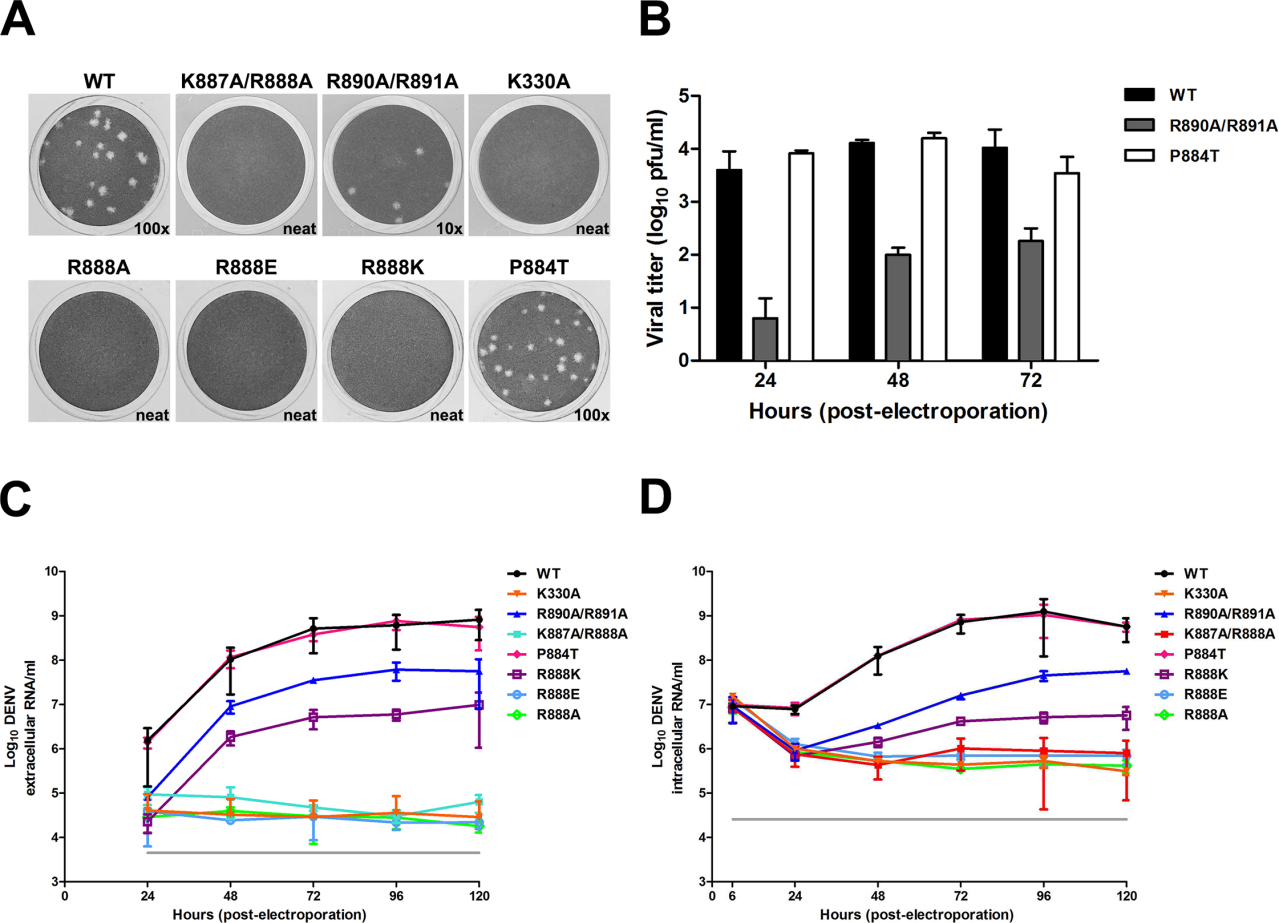
Role of Cter_18_ NS5 examined in a DENV2 infectious clone. **(A-D)** BHK-21 cells were electroporated with 10 μg of genomic-length RNA of WT DENV2 and mutants; supernatants and infected cells were harvested daily and consecutively for 5 days. Supernatants were used to **(A and B)** check for plaque production on BHK-21 cells and to **(C)** determine absolute copy numbers of extracellular viral RNA by real-time RT-PCR. Absolute copy numbers of viral RNA in log scale per ml of supernatant used for real-time RT-PCR was plotted; data are shown as the mean ± SD from two independent experiments. Data was normalised by deriving the absolute copy numbers from the mean values of the standards used in the two independent experiments. **(D)** RNA was extracted from infected cells and absolute copy numbers of intracellular viral RNA was determined by real-time RT-PCR. Absolute copy numbers of viral RNA per μg of RNA used for real-time RT-PCR was plotted; data are shown as the mean ± SD from two dependent experiments. The mutants were examined in two separate experiments. K887A/R888A, R890A/R891A and K330A were done in one set of experiment (n = 2) whereas R888A, R888E, R888K and P884T were done in a separate experiment (n = 2). WT was included in both experiment and only one set of WT data was shown here as representative of two independent experiments.

Overall the structural studies and the subcellular localization studies imply that the conformational presentation of the NS5 C-terminal residues determines their subcellular localization during the course of infection. Since we identified the Cter_18_ of DENV NS5 to be the key region for differential subcellular localization we embarked on testing the importance of the NLS binding determinants identified in the structure in the context of virus infection. We introduced K887A/R888A or R890A/R891A into the DENV2 cDNA clone, as previously described [17]. *In vitro* RNA transcripts that were produced from these clones were used to transfect BHK-21 cells and samples were harvested over the course of 5 days to assess viral replication by real-time qRT-PCR and NS5 localization by IF. No infectious virus was recovered from the supernatant of DENV2 NS5 K887A/R888A mutant virus and a ~ 2 log lower viral plaque was observed for DENV2 NS5 R890A/R891A compared to WT virus for day 2 and 3 post-transfection. Furthermore, a smaller plaque phenotype was observed for the latter (Fig 7A and 7B). To confirm that no infectious virus was present in the supernatant due to differential plaque morphologies, we carried out real-time qRT-PCR to measure absolute copy numbers of extracellular viral RNA. In agreement with the plaque assay data, the extracellular viral RNA level for DENV2 NS5 K887A/R888A mutant virus mirrored the previously published non-replicative control DENV2 NS5 K330A and remained consistently negative throughout the 5-day experiment, thus indicating no infectious virus particles were being produced for these mutant viruses (Fig 7C). Next, we also measured the copy numbers of both positive- and negative-sense intracellular RNA as before [17] and showed that the total intracellular viral RNA copy numbers of the DENV2 NS5 R890A/R891A mutant virus was at least 10-fold lower than DENV2 WT for days 1–4 post-transfection (Fig 7D). The intracellular viral RNA copy numbers for DENV2 NS5 K887A/R888A mutant virus and the non-replicative control NS5 K330A remained unchanged, implying that was no active RNA replication and the mutation was lethal to virus. In order to demonstrate that the defect in viral replication is not due to the enzymatic role of NS5 in viral replication, we purified both bacterially expressed N-terminal His_6_-tagged truncated DENV2 NS5 protein encompassing residues 1–882, or full-length NS5 protein that contained either K887A/R888A or R890A/R891A mutation for *de novo* initiation/elongation and primed elongation assays [34] (S5 Fig). The proteins were purified to >95% as determined by SDS-PAGE (S5A Fig) and were found to be similarly folded based on similar Tm values as determined by thermofluor assay (S5A Fig, top panel). As shown in S5B and S5C Fig, both K887A/R888A and R890A/R891A mutants and C-terminal truncated NS5 protein exhibited RdRp activity similar to WT NS5 protein. The data collectively suggests that residues 883–900 of NS5 do not influence the *in vitro* RdRp activity and suggests that the C-terminal region of NS5 may play an important structural role in viral replication within the RC in an infected cell.

**Fig 8:**
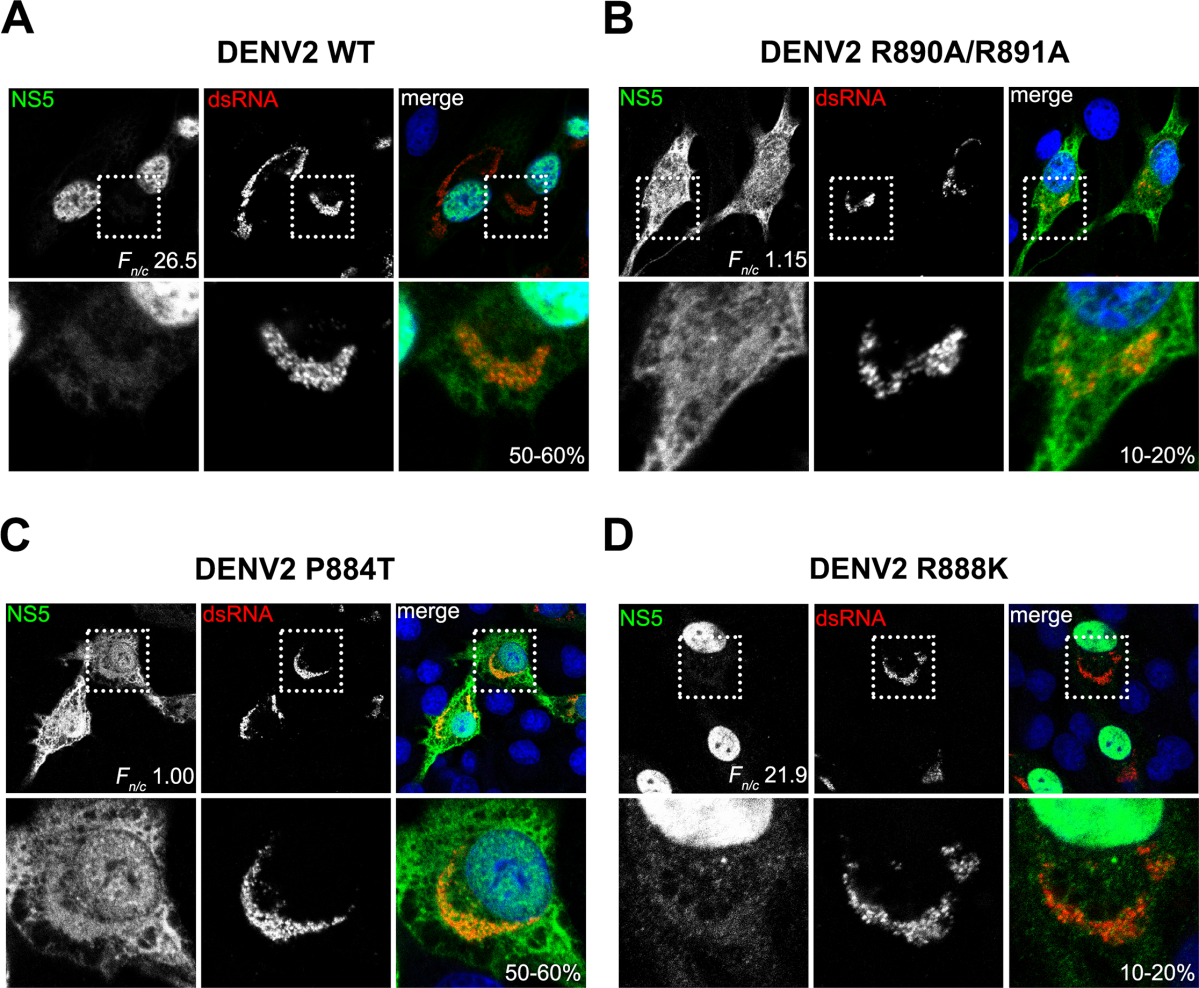
Polyphenotypic distribution of NS5 and dsRNA in wild type and mutant DENV in transfected cells. BHK-21 cells transfected with (A) DENV2 WT, (B) DENV2 R890A/R891A (C) DENV2 P884T and (D) DENV2 R888K (as in Fig 7) were analysed for presence of NS5 (green) and dsRNA (red) by IFA on day 3 post-transfection. Digitized images were captured by Zeiss LSM 710 upright confocal microscope by 63× oil immersion lens and image analysis was performed on with ImageJ software [52] to determine the nuclear to cytoplasmic fluorescence ratio (*F*_*n/c*_) of NS5 as done previously [17, 29, 30, 42]. The mean *F*_*n/c*_ ± SEM was calculated for ≥ 30 cells and the *F*_*n/c*_ values together with % of infectivity are indicated. Data from one experiment are shown. Insets: zoom-in views of the dotted boxed regions.

We next examined the subcellular localization and level of dsRNA for DENV2 WT and R890A/R891A mutant in transfected BHK-21 cells by CLSM. We observed the nuclear localization of DENV2 WT NS5 protein as earlier, and the distribution of DENV2 NS5 R890A/R891A mutant protein was similar to DENV1 NS5 and mainly in the cytoplasm. Notably, despite the high percentage of NS5 being localized to the nucleus in DENV2 WT transfected cells, we are able to detect the presence of NS5 in the cytoplasm (green, top row) which co-localizes well with the cytoplasmic dsRNA staining (red, middle row) on day 3 post-transfection (Fig 8). The level of dsRNA is more scattered and lower in the DENV2 NS5 R890A/R891A mutant even though much higher level of NS5 is present in the cytoplasm (Fig 8, right panel). This data suggests that only a fraction of total NS5 in the infected cell is needed for the viral RNA replication in the membrane associated replication complex within the cytoplasm and that the majority of the protein is shunted to the nucleus or other subcellular locations.

To explore the lethality involved with mutations in the basic residues that form the monopartite NLS within Cter_18_,in more detail we mutated DENV2 NS5 P884 to Thr (as found in DENV1 at position T883) in order to delineate the importance of presentation of the NLS for importin binding and its impact on infectivity and viral RNA replication. We also made a single mutation at DENV2 NS5 R888 to alanine, lysine or glutamate because this residue is completely conserved in all flaviviruses whilst K887 is not. Like the DENV2 NS5 K887A/R888A, the R888A and R888E mutants were completely non-viable (Fig 7A, 7C and 7D). R888K however was severely attenuated and no plaques could be observed although more than 2 log reduction in extracellular RNA could be detected by qRT-PCR. Interestingly, DENV2 NS5 P884T replicated like WT DENV2 NS5 but it showed a conspicuous difference in NS5 subcellular localization (Figs 7 and 8) similar to the data obtained for GFP-NS5 constructs bearing the same mutations (Fig 6A). Remarkably, the single amino acid change from Pro (as found in DENV2, 3 and 4) to Thr (as found in DENV1) at this position resulted in a more cytoplasmic DENV2 NS5, but yet its replication is still similar to WT DENV2 NS5. Taken together, the mutations in the C-terminal region in the DENV2 infectious clone suggest that R888 has a critical role in replication initiation that is independent of the subcellular distribution of NS5 but the C-terminal region may have an essential role in early events of RNA replication analogous to that of the HCV C-terminal peptide [41].

## Discussion

### The Cter_18_ of DENV1-4 NS5 defines its polyphenotypic sub-cellular localization

The elucidation of both DENV3 NS5 RdRp and the more recent full length NS5 structures reveal that NS5 residues 320–405 form an integral part of the RdRp domain, and are distributed within α2-α7 helices [13]. The α2-α4 helices (bNLS residues 320–341) form part of the thumb subdomain; α5 and α7 helices (residues 349–355 and 397–405 respectively) are found at the finger tips, between the fingers and thumb subdomains and α6 helix (residues 367–386) forms part of the palm subdomain. The a/bNLS (α6-α7) is thus distributed between the fingers and palm subdomains. In conjunction with biochemical and reverse genetics studies, NS5 residue K330, which occurs immediately N-terminal to α3, has been unambiguously identified as being critical for NS3 binding and viral RNA replication [26]. Furthermore, structures of full length NS5 [34] revealed that residue R352, which was also thought to be part of the bNLS, makes extensive interactions with residues in the MTase domain of NS5 and these interdomain interactions have now been shown to be functionally critical for non-enzymatic roles of NS5 in virus RNA replication and infectivity [34]. Taken together and given the location of bNLS and a/bNLS, it is not surprising that mutations [29, 30] or truncations [13, 42] in residues 320–405 destabilize the NS5 protein structure and can conceivably result in a pleiotropic effect on NS5 activity, nuclear localization and RNA replication. Therefore, from a structural perspective the residues 320–405 of DENV2 NS5 are unlikely to direct subcellular localization. This notion is further supported by analysis of the nuclear localization ability of residues DENV 2 NS5 residues 368–405 fused to 2xGFP (S6 and S7 Figs) in comparison with the newly discovered C-terminal 18 residues or the SV40 T antigen sequence.

The investigation into the distinctly different subcellular localization of DENV1 and DENV2 NS5 using gene swapping and CLSM quantification led to the surprising finding that the C-terminal 18 residues from 883–900 of NS5 is sufficient to determine its nuclear or cytoplasmic localization. This region does not appear to be necessary for *in vitro* RdRp activity (S5 Fig) and is not visible in most of the NS5 structures reported [11, 13, 34, 35]. The importance of the region in subcellular localization is supported by: (i) truncation of the C-terminal end of DENV2 NS5 by removing the last 18 amino acid residues, greatly reduced the nuclear targeting ability of NS5 (Fig 3B) despite the presence of an intact a/bNLS [29, 30], (ii) replacement of the C-terminal sequence of DENV1 NS5 with the corresponding region of DENV2 NS5, resulted in the protein being predominantly localized in the nucleus compared to WT DENV1 NS5 which is usually distributed between the cytoplasm and nucleus (Fig 3B), (iii) mutation of DENV2 NS5 C-terminal residues through alanine replacement or nonsense mutation resulted in a predominantly cytoplasmic NS5 protein (Fig 3A and 3B) and (iv) a chimeric protein construct of the C-terminal region of DENV2 NS5 (D2_883-900_) with a tandem GFP gene (2×GFP) was targeted to the nucleus (S8 Fig) to the same extent as the classical monopartite SV40 NLS compared to 2x GFP alone which was predominantly cytoplasmic.

However, one of the puzzles was that even though the binding determinants of Cter_18_ of DENV2 and 3 NS5 that bound to Impα are similar to Cter_18_ of DENV1 NS5, the binding affinity of the latter for the nuclear transport factor was weak (Fig 4). This prompted us to hypothesize that Thr883 (analogous positions P884 in DENV2,3 &4) may not constrain the presentation of DENV1 NS5 Cter_18_ for binding to Impα as might Pro in the same position. Indeed CLSM studies confirmed this notion: GFP-DENV1 NS5 T883P with presumably restricted presentation of the Cter_18_ became enriched mainly in the nucleus since the binding determinant is present in this sequence. In stark contrast GFP-DENV2 NS5 P884T showed the opposite phenotype and became predominantly cytoplasmic (Fig 6). More importantly, even in the context of DENV2 infectious clone the NS5 P884T mutation resulted in NS5 being mostly localized to the cytoplasm demonstrating that a single amino acid change can result in altered phenotype. Our data demonstrates that Cter_18_ of DENV2 and 3 NS5s may directly interact with cellular importins to target the protein to the nucleus while the Cter_18_ of DENV1 NS5 may have evolved to bind to other host factors as well and is mostly located in the cytoplasm. The Cter_18_ of DENV4 NS5 has the conserved Lys at P2 position found in NLSs but lacks other binding determinants needed for *in vitro* binding to Impα. However it has been predicted that Ser894 within Cter_18_ of DENV4 may be phosphorylated prior to shuttling to the nucleus (Figs 1 and S8) (40). This has not been formally shown in the context of infectious virus although computational modelling of Cter_18_ of DENV4 NS5 phosphorylated at Ser894 suggests it may interact with residues in Impα (S9 Fig). This requires further validation. Furthermore the mostly cytoplasmic location of DENV2 NS5 P884T makes it a valuable mutant to explore in detail the non-replication functions of NS5 that is involved in the modulation of innate immune response and other host factor interactions that may contribute to dengue pathogenesis [20–24].

Taken together, these findings demonstrate that the sequence directing the subcellular localization of DENV NS5 occurs within residues 883–900. The NS5 structural studies highlighted the challenges for residues 320–405 to form the signal sequence although the thumb subdomain has been identified as a “hot spot” for protein interactions [17, 26, 34].

### Structural determinants of polyphenotypic DENV NS5 subcellular localization

In this study the structures of Impα bound to DENV2 and 3 C-terminal NLS regions of NS5 provided a structural basis for differential nuclear localization. The DENV2 and 3 C-terminal 18 residues follow the consensus XXKR(^DE)(R/K), and is a class 2 NLS responsible for the nuclear localization of the NS5 [43]. DENV1 and 4 do not contain the same class 2 NLS which further suggests this region may be responsible for the differential subcellular localization observed within dengue serotypes. The two NS5 peptides bind the major binding site (ARM repeats 2–4) of Impα comprised of four principal binding cavities that interact with the NLS side-chain residues P2-P5 (Fig 5A and 5B). For all characterised NLSs, the most crucial structural determinant is a Lys residue located at position P2 [44]. The P2 Lys side-chain forms a salt bridge with the highly conserved importin-α Asp192 side-chain (Table 2). Although there is an observed preference for long basic amino-acids at the other major binding site positions (P3, P4, P5), NLSs can have a range of different amino-acids in these positions. The conserved Lys residue in the P2 major binding site groove is maintained in the in Cter_18_ of DENV1-4, and other NLS structures deposited to the PDB (Table 3). Overall, the importin-α recognition site used by the C-terminal region of NS5 to mediate nuclear localization is highly conserved, and the same site used by other cellular NLSs. Functional studies revealed that the highest affinity of Impα is to the C-terminal region of DENV2 and 3 NS5 which may be the driver for their prominent nuclear localization compared to DENV1 and 4. Complex structures of Impα and DENV2 and 3 C-terminal NLS region of NS5 provide a structural basis that the classical monopartite KRFR motif in the C-termini of DENV2 and DENV3 NS5 is responsible for nuclear localization.

**Table 3:** Comparison of PDB deposited NLSs binding only the Impα2 major site.

Source protein	IMPα2 Major cNLS binding site					PDB code
	P1	P2	P3	P4	P5	
Dengue 2 NS5 C-terminus	M	K	R	F	R	5HHG (in this paper)
Dengue 3 NS5 C-terminus	M	K	R	F	R	5FC8 (in this paper)
SV40T	K	K	K	R	K	1EJL [60], 1BK6 [61], 1Q1S [62], 1Q1T [62]
Human androgen receptor	R	K	L	K	K	3BTR [63]
αIBB	L	K	K	R	N	1IAL [50], 1IQ1 [64]
Venezuelan Equine Encephalitis Capsid	A	K	K	P	K	3VE6 [65]
Ku70	S	K	R	P	K	3RZX [66]
Ku80	A	K	K	L	K	3RZ9 [66]
CLIC4	A	K	K	Y	R	3OQS [67]

### Arginine 888 is critical for flaviviral RNA replication

**Fig 9:**
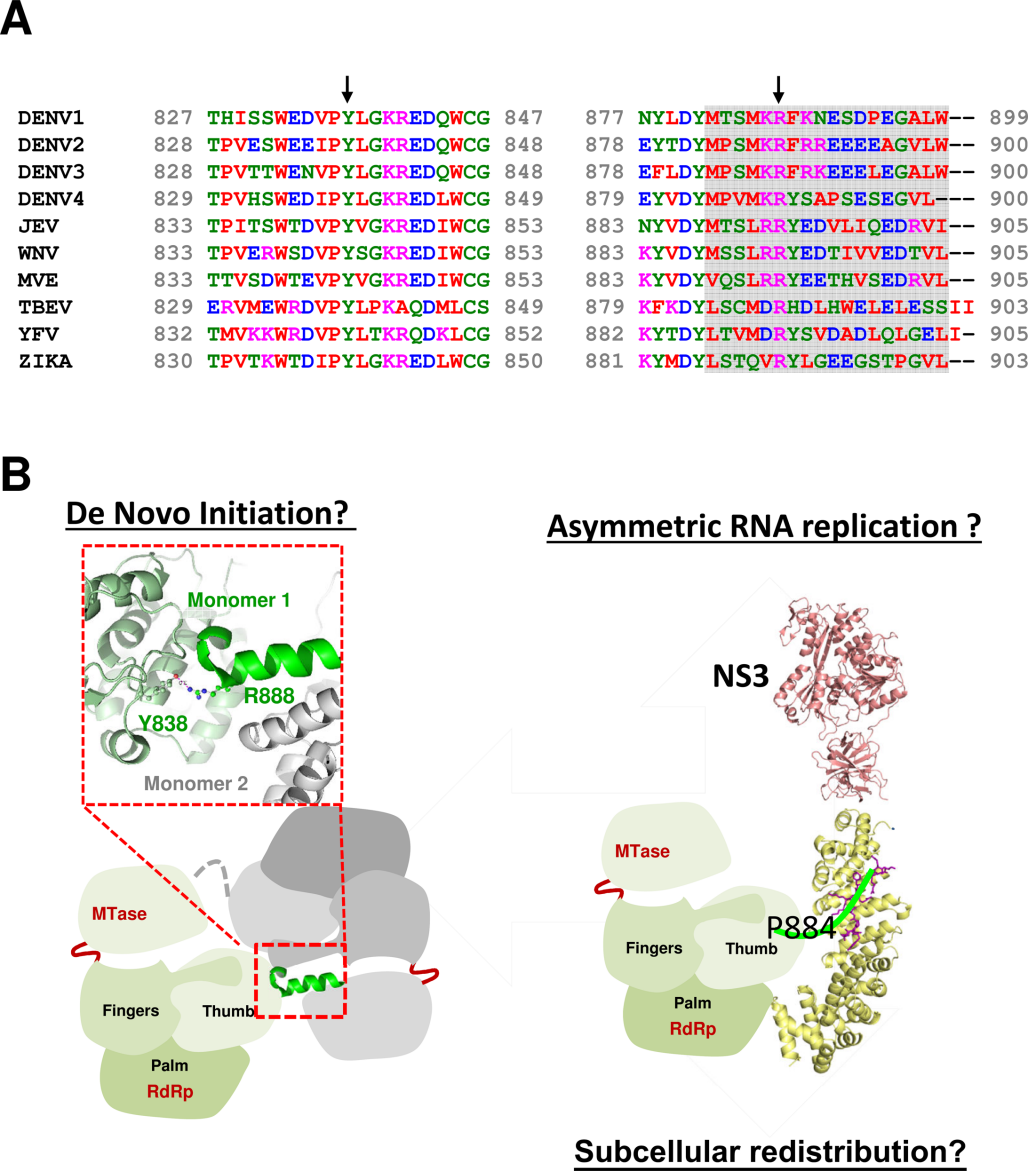
Sequence variation and conformational flexibility of NS5 Cter18 regulates the oligomeric state, nuclear localization, viral replication. **(A)** Sequence variation of residues 828–848 and 883–900 of DENV1–4 and representative flaviviruses. Arrow highlights the completely conserved Y838 and R888. The alignment was performed using Clustal Omega. The virus sequences and their GenBank accession numbers are as follows: DENV1-4 (same GenBank accession number as above), Japanese Encephalitis virus (JEV; M55506), West Nile virus (WNV; M12294), Murray Valley Encephalitis virus (MVE; AF161266), Tick-Borne Encephalitis Virus (TBEV; U27495), Yellow Fever virus (YFV; X15062) and Zika virus (ZIKV;KU497555) [58]. **(B)** Model of the role of the C-terminal region of NS5 that is required for the formation of dimer and the initiation of *de novo* viral RNA replication and interaction with importin-α or NS3. On the left: *de novo* RNA synthesis by NS5 oligomers independent of NS3 interaction as implied by NS3 N570A mutation [59] and non-viability of NS5 R888A as identified in this study. On the right: NS3 interaction with NS5 thumb domain in the RC will support higher plus strand RNA synthesis or NS5 Cter_18_ interacts with host importins or other factors to be localized to nucleus or cytoplasm in a regulated manner.

Although >95% of the NS5 is in the nucleus of DENV2 infected cells, the images obtained in this study clearly show co-localization between NS5 and dsRNA clustered in the perinuclear region (Fig 8A). Similarly for DENV1 NS5 where a large proportion of the protein is distributed in the cytoplasm, co-localization between NS5 and dsRNA in the perinuclear region is similarly confined to a specific area. These images suggest that for RNA replication to occur in a coordinated fashion within the replication complex, it may be necessary to limit the level of NS5 in this environment. In this respect, it is possible that the Cter_18_ of DENV1-4 NS5s may have undergone adaptive evolution to engage specific cellular proteins to shuttle the excess NS5s away from the replication factories. The fully functional DENV2 P884T mutant and the lethality of R888A mutation support this notion and suggests that the C-terminal region plays essential roles in the very early event in viral RNA replication and are independent of their role in nuclear localization (Figs 8C and S10). Indeed residue R888 is conserved in all four dengue serotypes at this position and also completely conserved in all flaviviruses including Zika virus [45] and Tick-Borne Encephalitis Virus NS5 (Fig 9A). So what is the role of R888 and how can it impact viral RNA replication?

One possibility is that it may be involved in an RNA binding event at the very early stage of viral RNA replication that is conserved in all flaviviruses. Mutational study on basic residues of NS5 have identified dibasic residues on the thumb subdomain, namely K840/R841 (located within helix α26), which are lethal to virus when mutated to alanine, despite an intact polymerase activity that can use viral RNA as template [46]. Based on the importance of the K887/R888 in this study and its location in thumb subdomain, it is tempting to hypothesize that R888 may be involved in binding to RNA for a critical RNA replication initiation event.

A second possibility that is supported by available NS5 structures and functional studies in the present work is that R888 may be involved in the oligomerization of NS5 which may be required for *de novo* RNA replication. This notion is based on the recently reported DENV3 NS5 structure carrying a six-residue priming loop deletion (^795^WSIHAH^800^) which crystallized as dimers [36] and revealed that the C-terminal region of NS5 can interact with neighbouring NS5 monomers. In the “type 2 dimers” where the C-terminal region could be resolved, residues R890 to G897 from one monomer formed an α-helix that interacts with the MTase domain of another NS5 molecule. Importantly R888 in the “type 2 dimer” makes a key interaction with the hydroxyl group of Y838, which is also completely conserved in all flaviviruses and is located in a loop between α25 and α26 in the same monomer (Fig 9B). It is likely that critical interactions for de novo priming are correctly positioned when R888 bonds with Y838 when NS5 oligomerizes during early stages of the infection. The survival of the virus is dependent on successful translation of the genomic RNA that is released from the infecting virion and the copying of negative-strand RNA template. Once sufficient negative-strand RNA is made, NS5 interacts with NS3 to carry out further synthesis of genomic RNA for packaging. Previously we showed that N570A mutation in NS3 permits negative-strand RNA synthesis in the early stages of infection but the weakened NS3-NS5 interaction prevents further new synthesis of positive-strand genomic RNA required for packaging the infectious virion. The NS5 R888A and R888E mutations are lethal in the infectious clone, whilst R888K appears to be slightly viable and infectivity was around 10% compared to WT and is able to support some negative-strand RNA synthesis compared to the former (S11 Fig). It is tempting to propose that the accumulation of negative-strand RNA in the first 3–12 hour post infection requires NS5 oligomerization. At the later stages NS5 interaction with NS3 and other unknown factors within the RC may require that the concentration of NS5 is limited and this is achieved by the excess NS5 being shunted to different subcellular locations using sequence features on the exposed C-terminal region to either the nucleus or throughout the cytoplasm away from the RC (Fig 9B). While this manuscript was under review, independent experimental support for the crucial role of Y838 in replication initiation was published in a study by Hodge and colleagues [47] using yeast three hybrid RNA binding to DENV RdRp as well as site directed mutatgenesis studies in a DENV replicon. They showed that the 3’UTR panhandle structure interacts near the thumb subdomain of RdRp in a region that contains several positively charged residues and also including the conserved Y838 that has been implicated in this work to be involved in RNA interactions together with R888. Based on RNA binding and mutational studies the authors suggested that the stacking interaction between the sidechain of Y838 and the RNA base may be required for replication initiation. Taken together, the C-terminal region which is located in the thumb subdomain along with the priming loop (emanating from α-helices in the thumb loop) may hold the key to initiate *de novo* RNA synthesis.

In summary, we have identified a motif within the Cter_18_ of NS5 from DENV1-4 and possibly other flaviviruses as main determinant for differential subcellular localization and distribution away from the RCs. Similar to HCV, NS5 Cter_18_ of flaviviruses may be critical for the early events during RNA replication [41]. The residues R888 and Y838 that are found to interact with each other in DENV are conserved in all flaviviruses including Zika virus that is causing high numbers of children to be born with birth defects (microcephaly) as well as causing increasing number of cases with Guillain-Barré syndrome [45]. The present discovery may guide the design of a pan-flavivirus inhibitor.

## Materials and Methods

### Cell lines and virus strains

Huh-7 cells (ATCC) were cultured in DMEM medium (Gibco) supplemented with 10% FBS, 100 units/ml penicillin, and 100 μg/ml streptomycin (1% P/S). Vero (ATCC) and HEK293T (ATCC) cells were cultured in DMEM high glucose medium (Gibco) supplemented with 10% FBS. A549 cells (ATCC) were cultured in Ham’s F-12K medium (Gibco) supplemented with 10% FBS and 1% P/S. BHK-21 cells (ATCC) were cultured in RPMI1640 medium (Gibco) supplemented with 10% FBS and 1% P/S. All cells were maintained at 37°C, with 5% CO_2_. C6/36 cells (ATCC) were cultured in RPMI1640 medium supplemented with 25 mM HEPES, 10% FBS and 1% P/S at 28°C, in the absence of CO_2_.

The four DENV serotypes (GenBank accession numbers for the relevant sequences are: DENV1 (EU081230), DENV2 (EU081177), DENV3 (EU081190) and DENV4 (GQ398256) that were used in the study was grown in C6/36 cells and titered in BHK-21 cells before storage at -80°C. These viruses were isolated during a local dengue outbreak that occurred in 2005 as part of the Early Dengue infection and outcome (EDEN) study in Singapore [48].

### Plasmid construction

His_6_-tagged DENV2 NS5 bacterial expression constructs: DENV2 NS5 gene was amplified by PCR from DENV2 cDNA clone (GenBank accession number: EU081177) [17, 48]. The PCR fragment was cloned into pET28b (Novagen) that was pre-cut with *Nde*I and *Sac*I by recombination with CloneEZ PCR cloning kit (Genscript) according to manufacturer’s protocol.

GST-tagged DENV1-4 NS5 peptide bacterial expression constructs: DENV1-4 NS5 gene fragments that corresponded to residues 865–900 of DENV2 NS5 were ordered from Genscript and amplified by PCR. The PCR products were digested with *Bam*HI and *Eco*RI and cloned into pre-cut PGEX4T-1 vector.

GFP-tagged DENV1 and 2 NS5 mammalian expression constructs: DENV1 and 2 NS5 genes were amplified by PCR from DENV1 (GenBank accession number: EU081230) and DENV2 cDNA clone (same GenBank accession number as above) [17, 48], respectively. The PCR fragments were digested with *Xho*I and *Xma*I and cloned into pre-cut pEGFP-C1 vector (Clontech).

DENV1 and 2 GFP-NS5 domain-swapped and motif-swapped gene segments were generated by overlap extension PCR. DENV1 and DENV2 GFP-NS5 constructs (as described) were used as templates for overlap extension PCR. The PCR products were digested and cloned into pEGFP-C1 vector as described above.

2×GFP-tagged DENV1-4 NS5 mammalian expression constructs: To create a 2×GFP vector, GFP gene was amplified by PCR from pEGFP-C1 plasmid with the forward primer 5’-GTACAAGTCCGGAATGGTGAGCAAGGGCGAGGAGC-3’ and the reverse primer 5’-CGCCGCCTCGAGCCTTGTACAGCTCGTCCATGCCGAG-3’. The PCR product was digested with *BspE*I and *Xho*I and cloned into pre-cut pEGFP-C1 vector. DENV1-4 NS5 gene fragments that corresponded to residues 883–900 of DENV2 NS5 were amplified by PCR from DENV1-4 cDNA clones (GenBank accession numbers for the relevant sequences are: DENV1 (same GenBank accession number as above), DENV2 (same GenBank accession number as above), DENV3 (EU081190) [48] and DENV4 (GQ398256) [49]). The PCR products were digested with *Xho*I and *Xma*I and cloned into pre-cut 2×GFP vector. SV40_NLS_ gene fragment (peptide sequence: PKKKRKV) was also cloned similarly into 2×GFP vector to serve as a positive control for monopartite NLS.

The full list of primers used for all the above mentioned plasmid constructions is available upon request.

### Plasmid transfection

One day prior to transfection, 1.6×10^5^ Vero cells were seeded onto 12-well plate containing glass cover slips, and incubated overnight at 37^°^C with 5% CO_2_. The following day, Vero cells were transfected using Lipofectamine 2000 (Invitrogen) with 2 μg of plasmid per well respectively, according to the manufacturer’s protocol.

### Site-directed mutagenesis

Deletion and point mutation of NS5 basic residues to alanine in both DENV2 His_6_-tagged and GFP-tagged NS5 plasmids were done using QuikChange II XL site-directed mutagenesis kit (Stratagene), according to the manufacturer’s protocol. The full list of primers is available upon request. Mutations were confirmed by automated DNA sequencing.

pWSK29 D2 fragment 3 (refer to [17], see Fig 4) was subjected to site-directed mutagenesis to generate point mutation at residues 884, 887, 888, 890 and 891. Mutation in fragment 3 clone was confirmed by sequencing and was excised from the vector by *Xba*I and *Sac*I and cloned into pWSK29 D2 fragment 1+2 plasmid that was similarly cut with *Xba*I and *Sac*I [17].

### Bacterial protein expression and purification

His_6_-tagged DENV2 NS5 bacterial expression constructs were transformed into *Escherichia coli* (*E*. *coli*) BL21 CodonPlus (DE3)-RIL cells (Stratagene) for protein expression and purified as previously published [34]. Heat stability analyses by thermo-fluoresence were performed on purified proteins as described previously to check protein folding and stability [11].

GST-tagged DENV1-4 NS5 peptide bacterial expression constructs were transformed into *E*. *coli* BL21 (DE3)-pLysS cells (Novagen). From an overnight starter culture, 500 μl was used to inoculate each covered sterile 2 L baffled flask containing 500 ml expression media (1mM MgSO_4_, ampicillin (100 μg/ml), 5% 20x NPS, 2% 50x 5052, 1% Tryptone, 0.5% yeast extract). The sterile flask was incubated at RT on a platform orbital at 90 rpm for 24 h. The cells were then harvested via centrifugation at 18°C and 6000 rpm, and the cell pellet resuspended in GST buffer A. The resuspended cells were run on a 4–12% Bis-Tris gel to ensure overexpression of GST-tagged DENV NS5 peptide.

Purification of GST-tagged DENV1-4 peptide constructs in complex with mouse importin-α lacking the auto-inhibitory importin-β binding domain (Impα) [50] were performed using on-column methods. Firstly, the cell suspensions were subjected to two freeze/thaw cycles and the addition of 1 ml lysozyme (20 mg/ml) to lyse the cell wall, and addition of 10 μl DNase (5 μg/ml) to degrade the DNA and reduce the viscosity of the sample. The cell extract was centrifuged at 12,000 rpm for 30 min at 10°C to remove cell debris, and further clarified by passage of the supernatant through a 0.45 μm syringe filter.

The soluble extract was injected onto a GST Buffer A equilibrated GST-trap 5 ml column using a superloop at 0.5 ml/min to allow interaction of the with GST-trap column. Following injection, the column was washed with 10 column volumes of GST buffer A and purified Impα was injected into column using a superloop at 0.5 ml/min to allow interaction of Impα with bound GST-NLS. The GST-trap column was then further washed with GST buffer A before elution of the complex with GST buffer B containing 10 mM glutathione. Elution fractions were analysed by SDS-PAGE, and pooled. Removal of the GST affinity tag, a necessary procedure for crystallisation, was performed by adding 50 μl thrombin (0.5 units/μl) and incubated overnight at 4°C. Separation of the GST tag from the NLS-Impα complex was achieved by gel filtration before concentration to 7.5 mg/ml for DENV2 C-NLS and 16 mg/ml for DENV3 C-NLS. All concentrated samples were assessed for purity using SDS-PAGE.

### Generation of in vitro transcribed m7-capped DENV 5’UTR-core-3’UTR RNA template

The DENV2 RNA template used in *de novo* initiation/elongation assay comprised of the DENV2 5′-UTR sequence, 72nt of the capsid-coding sequence, followed by the 3′-UTR [34]. Both the 5′-UTR-core and 3′-UTR sequences were obtained by PCR from DENV2 cDNA clone (same GenBank accession number as above) [17, 48] and overlap PCR was performed to join the T7 promoter-5′-UTR-core PCR product to the 3′-UTR sequence. The final PCR product (DENV 5′-UTR-core-3′-UTR) was gel purified and was *in vitro* transcribed using the T7 mMESSAGE mMACHINE kit (Ambion) according to the manufacturer’s protocol, with slight modification. Briefly, the reaction mix contained 5.6 μl of linearized DNA (≤3 μg), 2 μl of rGTP, rCTP and rUTP, 0.4 μ1 of rATP, 3 μl of m7G(5')ppp(5')A RNA Cap Structure Analog (total 2.5 units), 1 μ1 RNase inhibitor, 2 μ1 T7 RNA polymerase and 2 μ1 10× reaction buffer. The reaction mix was initially incubated at 37°C for 30 min, and the reaction was spiked with an additional 1.8 μl rATP and 0.2 μl 10× buffer before further incubation at 37°C for 2 h. 1 μl of TURBO DNase was added and the reaction was incubated at 37°C for 15 min to remove the DNA template. Following the addition of 27 μl nuclease-free water and 30 μl lithium chloride, RNA was allowed to precipitate overnight at -80°C. After overnight incubation, the reaction mix was spun at 13,200 rpm at 4°C for 15 min to pellet the RNA. The supernatant was carefully removed and the RNA pellet was resuspended in 1 ml of 70% ethanol. The mixture was spun at 13,200 rpm at 4°C for 10 min and the 70% ethanol was removed. The RNA pellet was allowed to air-dry at RT and resuspended in RNase-free water. The RNA concentration was measured by NanoDrop 2000 UV-Vis Spectrophotometer. The resuspended RNA was run on 0.6% DNA agarose gel to check the RNA integrity.

### De novo initiation/elongation and elongation assay

The *de novo* initiation/elongation assay was carried out as previously described [34], with slight modification. Briefly, the reaction comprised of 100 nM DENV2 NS5 wild-type or mutant protein, 100 nM *in vitro* transcribed m7-capped DENV2 5´-UTR-core-3´-UTR RNA, 20 μM ATP, 20 μM GTP, 20 μM UTP, 5 μM Atto-CTP (Trilink Biotechnologies), in a volume of 15 μl of assay buffer (50 mM Tris-HCl, pH 7.5, 10 mM KCl, 1 mM MgCl_2_, 0.3 mM MnCl_2_, 0.001% Triton X-100 and 10 μM cysteine). The elongation assay was carried out as previously described [51], with slight modification. Briefly, the reaction comprised of 100 nM DENV2 NS5 wild-type or mutant protein, 100 nM pre-annealed DENV2 3′-UTR-U_30_ RNA (comprising a 5′ single-stranded sequence of U_30_ followed by a 10-nt duplex hairpin sequence from DENV2 3′-UTR; 5′-U_30_-AACAGGUUCU*AGAACCUGUU*-3′) [51], 5 μM Atto-CTP (Trilink Biotechnologies), in a volume of 15 μl of assay buffer (50 mM Tris-HCl at pH 7.5, 10 mM KCl, 0.5 mM MnCl_2_, 0.01% Triton X-100, and 10 μM cysteine). The DENV2 3′-UTR-U_30_ RNA was heated at 95°C for 3 min, and cooled to RT for pre-annealing. All reactions were allowed to proceed for up to 3 h at RT. At the indicated time-points, 10 μl of 2.5× STOP buffer (200 mM NaCl, 25 mM MgCl_2_, 1.5 M DEA, pH 10; Promega) with 25 nM calf intestinal alkaline phosphatase (CIP; New England Biolabs) was added to the wells of both assays to stop the reactions. The plate was shaken and centrifuged briefly at 1200 rpm, followed by incubation at RT for 60 min and the released AttoPhos was monitored by reading on a Tecan Infinite *F200* PRO microplate reader at excitation_max_ and emission_max_ wavelengths of 422 nm and 566 nm, respectively. All data points were collected in duplicates in 384-well black opaque plates (Corning).

### Confocal laser scanning microscopy and computation of nuclear to cytoplasmic fluorescence ratio (F_n/c_)

At an appropriate time post-transfection, cells that were either transfected with mammalian expression plasmids or *in vitro* transcribed viral RNA, or mock-transfected were fixed with ice-cold methanol: acetone (1:1) for 15 min at -20°C. Following fixation, cells were washed once by PBS and analyzed by immunofluorescence assay, using primary antibodies against GFP (ab6556, 1:1000; Abcam), or NS5 (5R3, 30 nM [33]), and secondary antibodies coupled to Alexa-Fluor 488 or Alexa-Fluor 594 (Invitrogen). Coverslips were mounted using ProLong Gold antifade reagent with DAPI (Invitrogen). Digitized images were captured by Zeiss LSM 710 upright confocal microscope (Carl Zeiss, Germany) at 40/64× magnification. Image processing was performed with ImageJ software [52]. For grey-scale image, the 8-bit full-colour image was converted to 16-bit grey-scale image.

Image analysis was performed on digitized images with ImageJ software [52] to determine nuclear to cytoplasmic fluorescence ratio (*F*_*n/c*_) of each cell using the following formula: *F*_*n/c*_ = (*F*_n_-*F*_b_)/(*F*_c_-*F*_b_), where *F*_n_ is the nuclear fluorescence, *F*_c_ is the cytoplasmic fluorescence and *F*_b_ is the background fluorescence [28–30, 42]. *F*_*n/c*_ value of > 1 indicates nuclear localization, < 1 indicates cytoplasmic localization and = 1 indicates equal distribution between the nucleus and cytoplasm. The mean *F*_*n/c*_ ± SEM was calculated for ≥ 30 cells.

### Microtitre plate binding assays

The wells of the glutathione coated plate (Thermo Scientific Pierce) were rinsed three times with 200 μl of wash buffer (PBS containing 0.05% Tween-20), followed by addition of 100 μl of each GST-NLS cell lysate in triplicate. The plate was covered and incubated for 1 h at RT and then washed a further three times with wash buffer. 100 μl of serially diluted Impα (2-fold serial dilutions in PBS, starting from 12.5 μM) was added to each respective wells, and incubation for 1 h at RT. The plate was then washed three times with wash buffer and 100 μl of the prepared anti-6x His tag HRP antibody (1/5000 dilution) added to each well. The plate was incubated in the dark for 1 h at RT, and washed three times with 200 μl/well of wash buffer. The binding reaction was initiated by the addition of 100 μl of 3,3′,5,5′-tetramethylbenzidine (TMB) substrate per well. After 20 min incubation, 100 μl of stop solution (1 M H_2_SO_4_) was added to stop the reaction, and measured using a plate reader at 450 nm. The K_D_ was calculated using the one-site specific binding analysis with Prism Graphpad 6.0f.

### Bead-binding assay

100 μl of clarified GST-NLS cell lysate was incubated with 50 μl of glutathione-agarose beads for 30 min at 4°C. Following incubation, the beads were washed three times in 1 ml of GST buffer A (50 mM Tris pH 8.0 and 125 mM NaCl) to remove unbound GST-NLS fusion protein. The bound beads were resuspended in 50 ml GST buffer A to obtain a 1 nM concentration of bound GST-NLS fusion protein. Serially diluted Impα (2-fold serial dilutions in GST buffer A, starting from 800 nM) was incubated with GST-NLS fusion protein bound beads for 30 min at 4°C. The beads were pelleted at 500 x g for 5 min and unbound Impα was removed. Bound Impα and GST-NLS fusion proteins were eluted by incubating beads with 100 μl of GST buffer B (50mM Tris pH 8.0, 125 mM NaCl and 10 mM reduced L-glutathione) for 10 min at RT. The beads were pelleted and the eluted proteins were analysed by SDS-PAGE. Densitometry and binding affinity analyses were performed using ImageJ and Prism Graphpad 6.0f, respectively.

In vitro transcription, RNA electroporation, plaque assay, real-time RT-PCR (reverse transcription-PCR) and immunofluorescence assay (IFA)

The above experiment was performed as per described previously [17]. IFA against NS5 protein by anti-NS5 human antibody 5R3 [34] and dsRNA by anti-dsRNA mouse antibody J2 (Scicons) was performed. IFA images were captured on inverted fluorescence microscope (Olympus IX71, Center Valley, USA) at 40x magnification and image analysis was performed with ImageJ software [52].

### Crystallization, data collection and structure solution of Impα-NLS complexes

Crystals of Impα in complex with DENV3 NS5 C-terminal NLS were obtained in a condition containing 1 M sodium citrate and 10 mM DTT at pH 7, and DENV2 NS5 C-terminal NLS were obtained in a condition containing 1 M ammonium sulfate and 0.1 M sodium HEPES at pH 7. X-ray diffraction data were collected at the Australian Synchrotron on the MX1 macromolecular crystal beam line [53]. Data were processed using iMosFlm [54], where the diffraction images were indexed and integrated, then scaled, phased and refined using AIMLESS [55–57], Phenix Phaser MR, and Phenix.refine, respectively [57]. Structural modelling was performed using COOT to ensure all molecules were correctly positioned within the electron density map generated from the collected X-ray data. Once the best fit between the structural model and experimental data was achieved (measured by R-free, R-work), and other model validation procedures confirmed that the model was of good quality, the model and data was deposited into the protein data bank (PDB) for final validation.

### Protein Data Bank deposition

All protein structure coordinates and structure factor files for DENV2 C-terminal NLS and DENV3 C-terminal NLS complexes with importin alpha are available from the Protein Data Bank database (accession numbers: 5HHG and 5FC8, respectively).

### Statistical analysis

Student’s t-test was used to determine statistical significance. P values ≤ 0.05 were considered as significant.

## Supporting Information

S1 Fig NS5 subcellular localization pattern in DENV infected A549 and Vero cells. **(A)** A549 cells and **(B)** Vero cells were infected with DENV1-4 at MOI 10 and the infected cells (>90%) were analysed for presence of NS5 (green) and dsRNA (red) by IFA at 24h post-infection. Digitized images were captured by Zeiss LSM 710 upright confocal microscope by 63× oil immersion lens. Image analysis was performed on digitized images of NS5 staining with ImageJ software [52] to determine nuclear to cytoplasmic fluorescence ratio (*F*_*n/c*_) as done previously [28–30,42]. The mean *F*_*n/c*_ ± SEM was calculated for ≥ 30 cells.
(TIF)

S2 Fig Sequence alignment of residues 883–900 of DENV1-4 NS5 with SV_40_ monopartite NLS. The alignment was performed using Clustal Omega. Basic amino acid residues similar to those of DENV2 NS5 (at position 887–888 and 890–891) in the aligned sequences are shaded in grey. The virus sequences and their GenBank accession numbers are as follows: DENV1 (EU081230), DENV2 (EU081177), DENV3 (EU081190) and DENV4 (GQ398256). The residue numbering is indicated above the alignment and it is based on DENV2 protein sequence.
(TIF)

S3 Fig Subcellular localization pattern of full-length and truncated DENV1 and DENV2, and chimeric full-length DENV1/2 GFP-NS5 in BHK21 and HEK293T cells. **(A and B)** GFP-NS5 protein constructs described in Figs 2A and 3A were transfected into **(A)** BHK21 cells and **(B)** HEK293T cells and fixed at 24-hour post-transfection. Anti-GFP (ab6556 IgG, 1:1000) antibody was used for immunostaining. Digitized images were captured by Zeiss LSM 710 upright confocal microscope by 40× oil immersion lens. The construct numbers used in Fig 2A are indicated in parenthesis in the images.
(TIF)

S4 Fig Crystals of DENV2 and DENV3 NS5 C-terminal NLS:Impα. **(A and B)** Gel filtration profiles of **(A)** DENV2 NS5 C-terminal NLS:Impα and **(B)** DENV3 NS5 C-terminal NLS:Impα. To obtain DENV-NLS:Impα complex, size exclusion chromatography was undertaken on AKTA FPLC using an S200 26/60 column (GE Healthcare). A protein sample of ≤ 12 ml was loaded into a column that was pre-equilibrated with GST buffer A at a flow rate of 2.5 ml/min. The larger DENV-NLS:Impα complex eluted before GST dimer and good separation was observed. Fractions containing pure DENV-NLS:Impα complex were combined, and concentrated using a 15 ml, 10 kDa Amicon centrifuge device as per manufacturer’s instruction. The final purified and concentrated protein DENV2 (7.5 mg/mL) and DENV3 (16 mg/mL) were put into crystal trials and yielded rod-shaped crystals. **(C and D)** Protein crystals in hanging drop. Rod-shaped crystals of Impα in complex with **(C)** DENV2 NS5 C-terminal NLS and **(D)** DENV3 NS5 C-terminal NLS were obtained in conditions containing 1 M ammonium sulfate and 0.1 M sodium HEPES at pH 7, and 1 M sodium citrate and 10 mM DTT at pH 7, respectively.
(TIF)

S5 Fig Biochemical characterization of RdRp activity of C-terminal truncated and mutated NS5 proteins. **(A)** His_6_-tagged NS5 proteins were expressed in E. coli and were purified from cell lysates by Ni-NTA affinity chromatography with HisTrap HP 1 ml column and size-exclusion chromatography with HiPrep Superdex-200 gel filtration column. 2 μg protein was analyzed for purity and integrity by SDS-PAGE and Coomassie blue staining. Numbers on the top are the melting temperature of purified proteins (Tm values) measured by thermofluor assay are indicated in parenthesis. Results are shown as the mean of duplicates from one independent experiment. Numbers on the left are the sizes of molecular mass standards in kDa. **(B and C)** RdRp activity of DENV2 WT and mutant NS5 proteins was measured in **(B)** *de novo* initiation/elongation and **(C)** elongation assays. **(B)** *De novo* initiation/elongation was carried out with 100 nM capped DENV 5’UTR-core-3’UTR RNA (corresponding to nucleotides 1 to 175 and 10277 to 10723 of the genome), 100 nM purified NS5 protein and 5 μM Atto-CTP. **(C)** Elongation assay was carried out 100 nM DENV2 3′UTR-U_30_ RNA (corresponding to nucleotides 10714 to 10723 of the genome), 100 nM purified NS5 protein and 3 μM Atto-ATP. **(B and C)** The amount of released AttoPhos in both assays was monitored by reading the reaction mix on a microplate reader at excitation_max_ and emission_max_ wavelengths 422 nm and 566 nm, respectively. The activity of each protein is expressed as percentage relative to the activity of WT NS5. Results are shown as the mean ± SD of duplicates from two independent experiments.
(TIF)

S6 Fig Subcellular localization pattern of DENV2 NS5 R888K in transfected BHK21 cells. BHK-21 cells transfected with DENV2 WT and DENV2 P888K were analysed for presence of NS5 (green) and dsRNA (red) by IFA on day 3 post-transfection. Digitized images were captured by Zeiss LSM 710 upright confocal microscope by 63× oil immersion lens and image analysis was performed on with ImageJ software to determine the nuclear to cytoplasmic fluorescence ratio (Fn/c) of NS5 as done previously. The mean Fn/c ± SEM was calculated for ≥ 30 cells and the Fn/c values are indicated. Data from one experiment are shown.
(TIF)

S7 Fig Fusion of residues 368–405 of DENV2 NS5 to 2×GFP does not target 2×GFP to the nucleus. Vero cells were transfected with either 2×GFP or 2×GFP-fusion plasmids encoding residues 320–405, 320–367, 368–405, 368–389, 387 and 405 and 883–900 of DENV2 NS5, and fixed at 24h post-transfection. 2×GFP-fusion plasmid encoding SV40 NLS was included as a positive control for monopartite NLS. The cells were stained and images were captured as described previously.
(TIF)

S8 Fig Functional characterization of DENV2 NS5 C-terminal monopartite NLS. Vero cells were transfected with either 2×GFP or 2×GFP-fusion plasmids, and fixed at 24h post-transfection. 2×GFP-fusion plasmid encoding SV_40_ NLS was included as a positive control for monopartite NLS. Anti-GFP (ab6556 IgG, 1:1000) antibody was used for immunostaining and digitized images were captured by Zeiss LSM 710 upright confocal microscope by 40× oil immersion lens. Nuclear to cytoplasmic fluorescence ratio (*F*_*n/c*_) as previously described [28–30,42] are indicated and data are shown as mean *F*_*n/c*_, n ≥ 30 cells from a single assay, representative of two independent experiments.
(TIF)

S9 Fig Structure based modelling of the DENV4 NLS peptide containing phosphorylated Ser894. **(A)** Impα is shown as yellow ribbons, with key interacting R101 residue shown in magenta, the backbone of DENV4 C-terminal NLS model is shown in blue and (B) as in A, with the phosphorylated serine shown in orange. The models were made using COOT and the simple mutate function on the structure of DENV3 C-terminal NLS in complex with Impα. The serine was phosphorylated using “the phosphorylate this residue function” in the modelling tools of COOT. The distance was measured using the measure function in COOT and images were created using PyMOL.
(TIF)

S10 Fig Subcellular localization pattern of DENV2 GFP-NS5 R888A in Vero cells. GFP, DENV2 GFP-NS5 WT and R888A protein constructs were transfected into Vero cells and fixed at 24 hr post-transfection. Anti-GFP (ab6556 IgG, 1:1000) antibody was used for immunostaining and digitized images were captured by Zeiss LSM 710 upright confocal microscope by 63× oil immersion lens.
(TIF)

S11 Fig Positive and negative strand synthesis in WT and mutated DENV2 infectious clone. BHK-21 cells were electroporated with 10 μg of genomic-length RNA of WT DENV2 and mutants; supernatants and infected cells were harvested daily and consecutively for 5 days [59]. RNA was extracted from infected cells and absolute copy numbers of intracellular viral positive (solid line) and negative (dotted line) RNA was determined by real-time PCR. The values of viral genome numbers were normalized to actin expression level and absolute copy number of viral RNA per μg of RNA was plotted; data are shown as the mean ± SD from two dependent experiments.
(TIF)
