# Supplementary figures and images for: The C-terminal 18 Amino Acid Region of Dengue Virus NS5 Regulates its Subcellular Localization and Contains a Conserved Arginine Residue Essential for Infectious Virus Production
Source: PLoS Pathog. 2016 Sep 13;12(9):e1005886. doi: 10.1371/journal.ppat.1005886 (PMC5021334; doi:10.1371/journal.ppat.1005886)

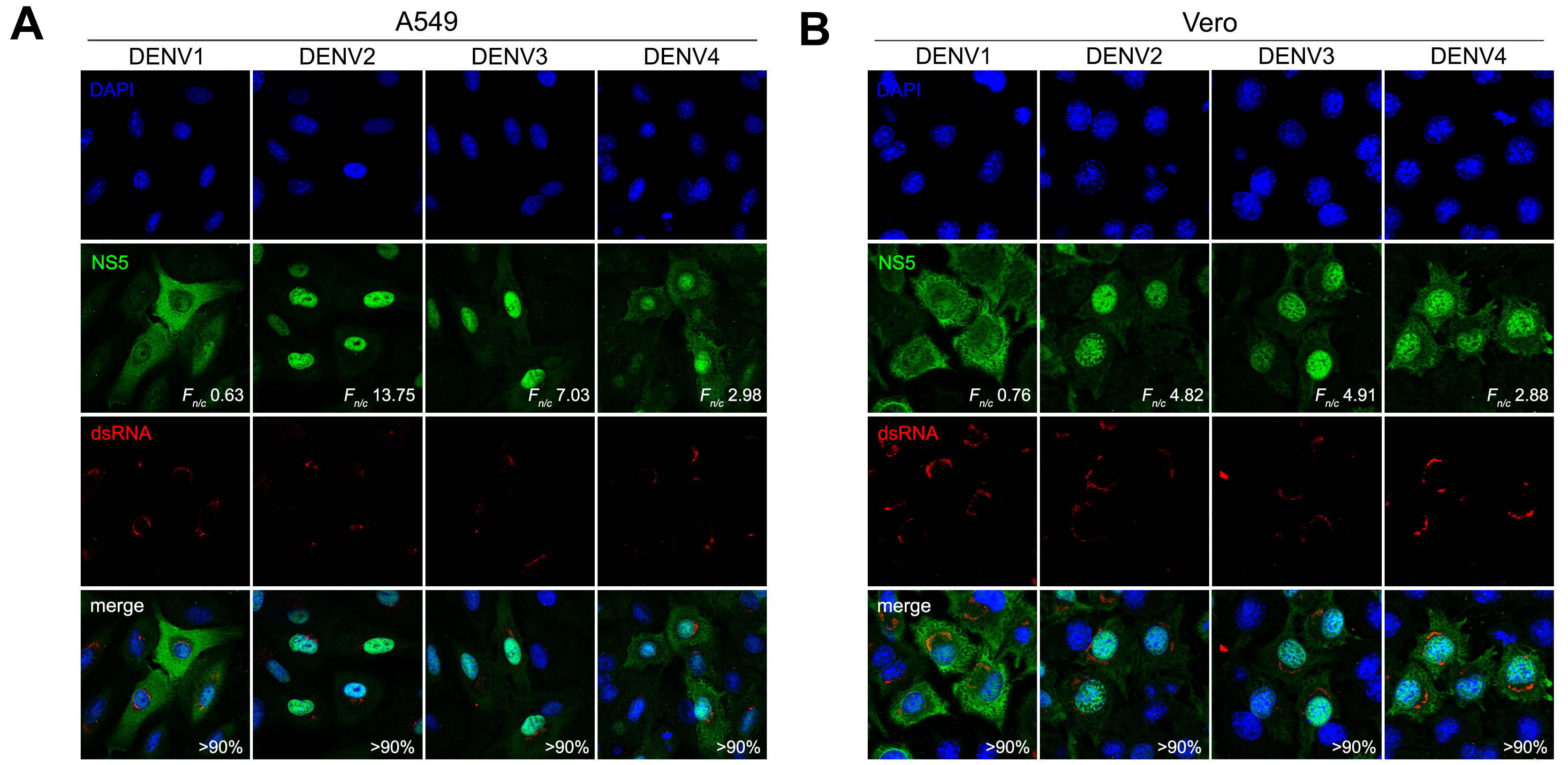

Supplement: S1 Fig — (A) A549 cells and (B) Vero cells were infected with DENV1-4 at MOI 10 and the infected cells (>90%) were analysed for presence of NS5 (green) and dsRNA (red) by IFA at 24h post-infection. Digitized images were captured by Zeiss LSM 710 upright confocal microscope by 63× oil immersion lens. Image analysis was performed on digitized images of NS5 staining with ImageJ software [52] to determine nuclear to cytoplasmic fluorescence ratio (F n/c) as done previously [28–30,42]. The mean F n/c ± SEM was calculated for ≥ 30 cells. (TIF) [file ppat.1005886.s001.tif]

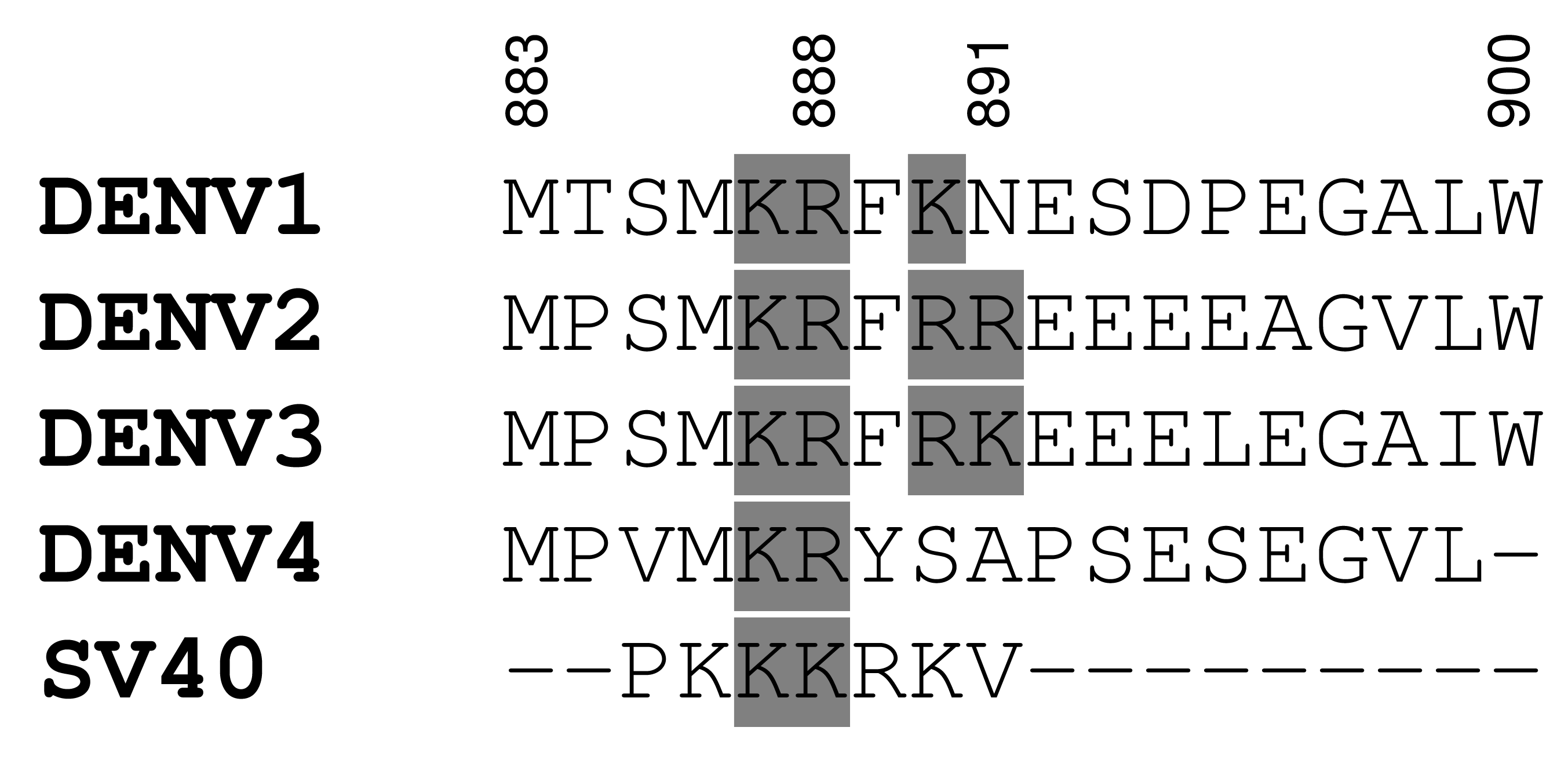

Supplement: S2 Fig — The alignment was performed using Clustal Omega. Basic amino acid residues similar to those of DENV2 NS5 (at position 887–888 and 890–891) in the aligned sequences are shaded in grey. The virus sequences and their GenBank accession numbers are as follows: DENV1 (EU081230), DENV2 (EU081177), DENV3 (EU081190) and DENV4 (GQ398256). The residue numbering is indicated above the alignment and it is based on DENV2 protein sequence. (TIF) [file ppat.1005886.s002.tif]

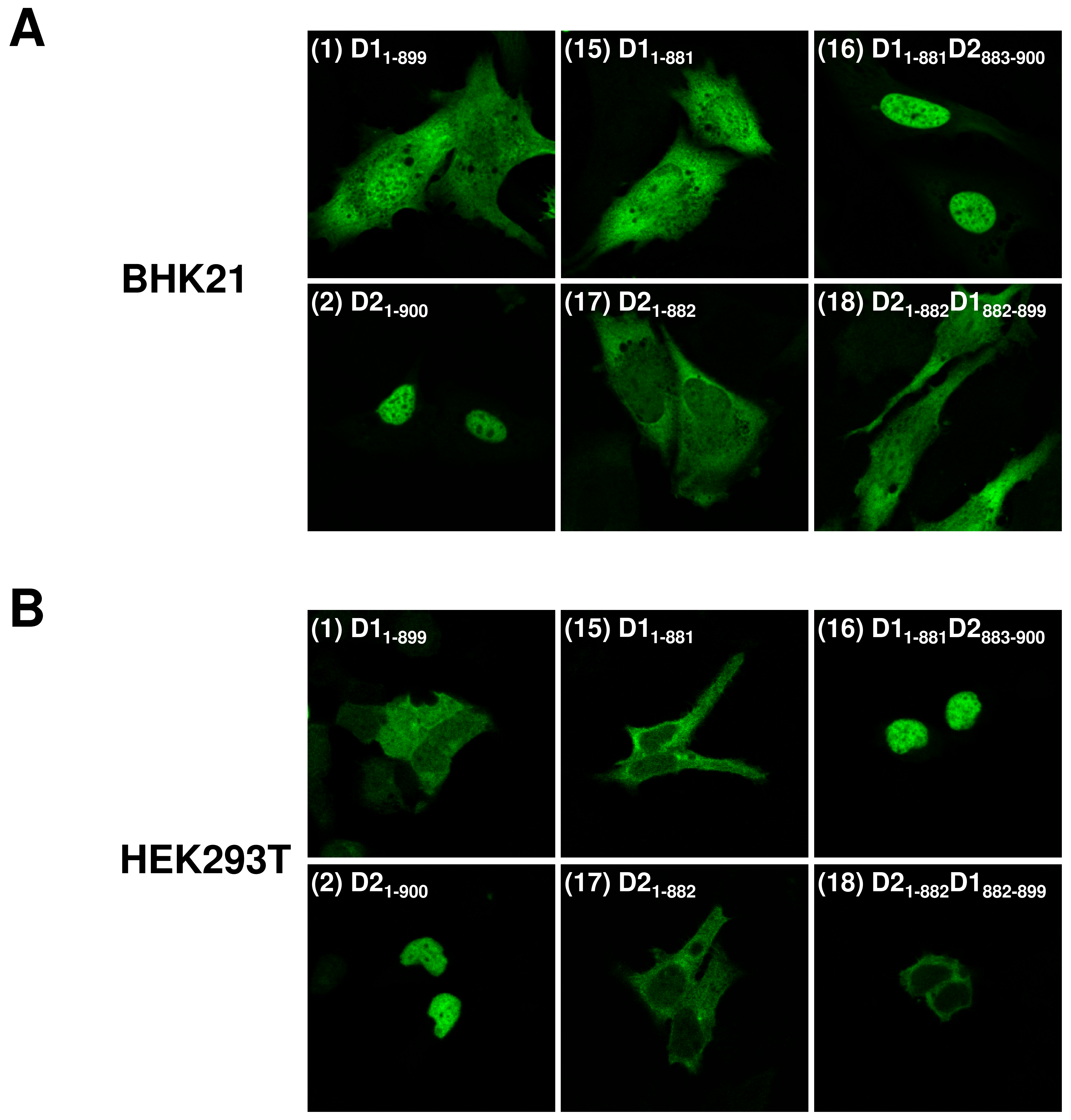

Supplement: S3 Fig — (A and B) GFP-NS5 protein constructs described in Figs 2A and 3A were transfected into (A) BHK21 cells and (B) HEK293T cells and fixed at 24-hour post-transfection. Anti-GFP (ab6556 IgG, 1:1000) antibody was used for immunostaining. Digitized images were captured by Zeiss LSM 710 upright confocal microscope by 40× oil immersion lens. The construct numbers used in Fig 2A are indicated in parenthesis in the images. (TIF) [file ppat.1005886.s003.tif]

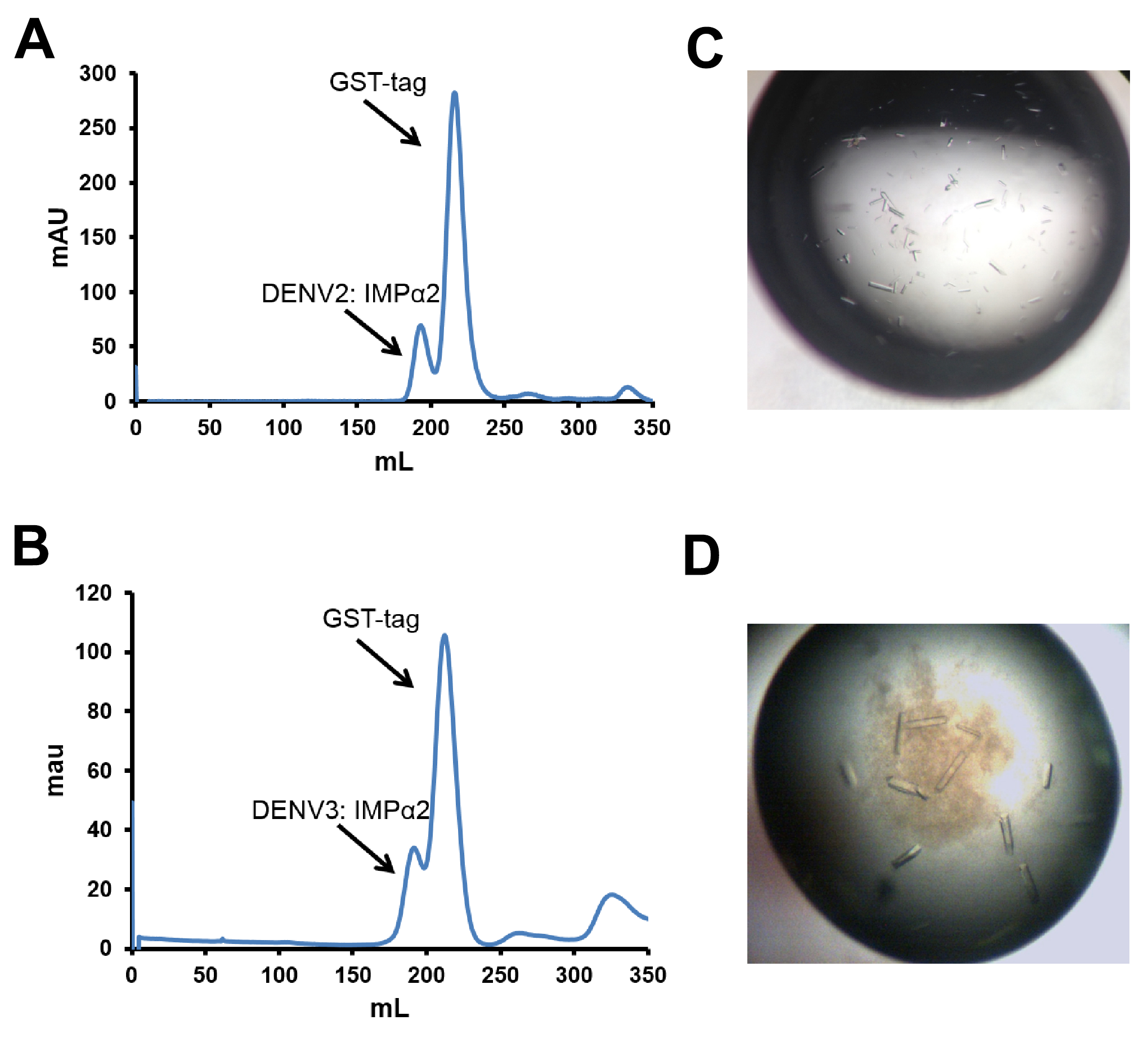

Supplement: S4 Fig — (A and B) Gel filtration profiles of (A) DENV2 NS5 C-terminal NLS:Impα and (B) DENV3 NS5 C-terminal NLS:Impα. To obtain DENV-NLS:Impα complex, size exclusion chromatography was undertaken on AKTA FPLC using an S200 26/60 column (GE Healthcare). A protein sample of ≤ 12 ml was loaded into a column that was pre-equilibrated with GST buffer A at a flow rate of 2.5 ml/min. The larger DENV-NLS:Impα complex eluted before GST dimer and good separation was observed. Fractions containing pure DENV-NLS:Impα complex were combined, and concentrated using a 15 ml, 10 kDa Amicon centrifuge device as per manufacturer’s instruction. The final purified and concentrated protein DENV2 (7.5 mg/mL) and DENV3 (16 mg/mL) were put into crystal trials and yielded rod-shaped crystals. (C and D) Protein crystals in hanging drop. Rod-shaped crystals of Impα in complex with (C) DENV2 NS5 C-terminal NLS and (D) DENV3 NS5 C-terminal NLS were obtained in conditions containing 1 M ammonium sulfate and 0.1 M sodium HEPES at pH 7, and 1 M sodium citrate and 10 mM DTT at pH 7, respectively. (TIF) [file ppat.1005886.s004.tif]

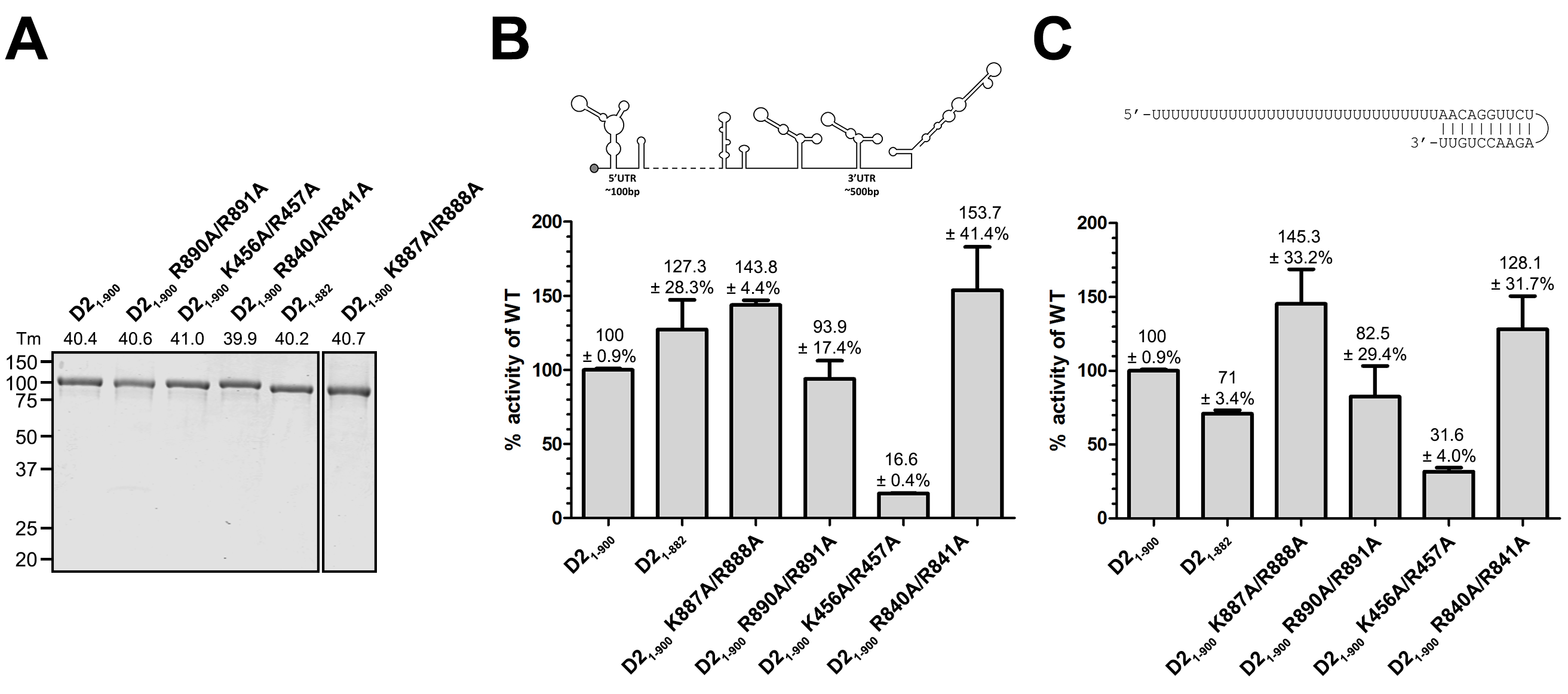

Supplement: S5 Fig — (A) His6-tagged NS5 proteins were expressed in E. coli and were purified from cell lysates by Ni-NTA affinity chromatography with HisTrap HP 1 ml column and size-exclusion chromatography with HiPrep Superdex-200 gel filtration column. 2 μg protein was analyzed for purity and integrity by SDS-PAGE and Coomassie blue staining. Numbers on the top are the melting temperature of purified proteins (Tm values) measured by thermofluor assay are indicated in parenthesis. Results are shown as the mean of duplicates from one independent experiment. Numbers on the left are the sizes of molecular mass standards in kDa. (B and C) RdRp activity of DENV2 WT and mutant NS5 proteins was measured in (B) de novo initiation/elongation and (C) elongation assays. (B) De novo initiation/elongation was carried out with 100 nM capped DENV 5’UTR-core-3’UTR RNA (corresponding to nucleotides 1 to 175 and 10277 to 10723 of the genome), 100 nM purified NS5 protein and 5 μM Atto-CTP. (C) Elongation assay was carried out 100 nM DENV2 3′UTR-U30 RNA (corresponding to nucleotides 10714 to 10723 of the genome), 100 nM purified NS5 protein and 3 μM Atto-ATP. (B and C) The amount of released AttoPhos in both assays was monitored by reading the reaction mix on a microplate reader at excitationmax and emissionmax wavelengths 422 nm and 566 nm, respectively. The activity of each protein is expressed as percentage relative to the activity of WT NS5. Results are shown as the mean ± SD of duplicates from two independent experiments. (TIF) [file ppat.1005886.s005.tif]

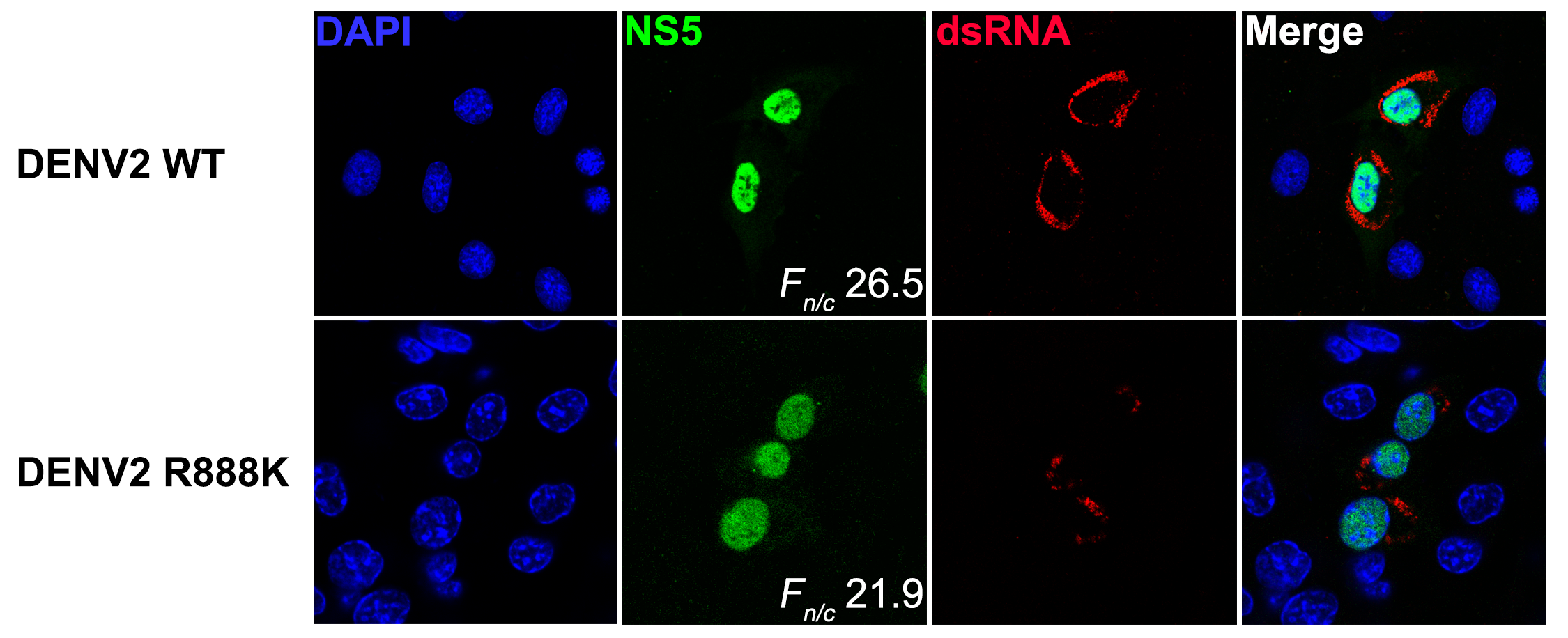

Supplement: S6 Fig — BHK-21 cells transfected with DENV2 WT and DENV2 P888K were analysed for presence of NS5 (green) and dsRNA (red) by IFA on day 3 post-transfection. Digitized images were captured by Zeiss LSM 710 upright confocal microscope by 63× oil immersion lens and image analysis was performed on with ImageJ software to determine the nuclear to cytoplasmic fluorescence ratio (Fn/c) of NS5 as done previously. The mean Fn/c ± SEM was calculated for ≥ 30 cells and the Fn/c values are indicated. Data from one experiment are shown. (TIF) [file ppat.1005886.s006.tif]

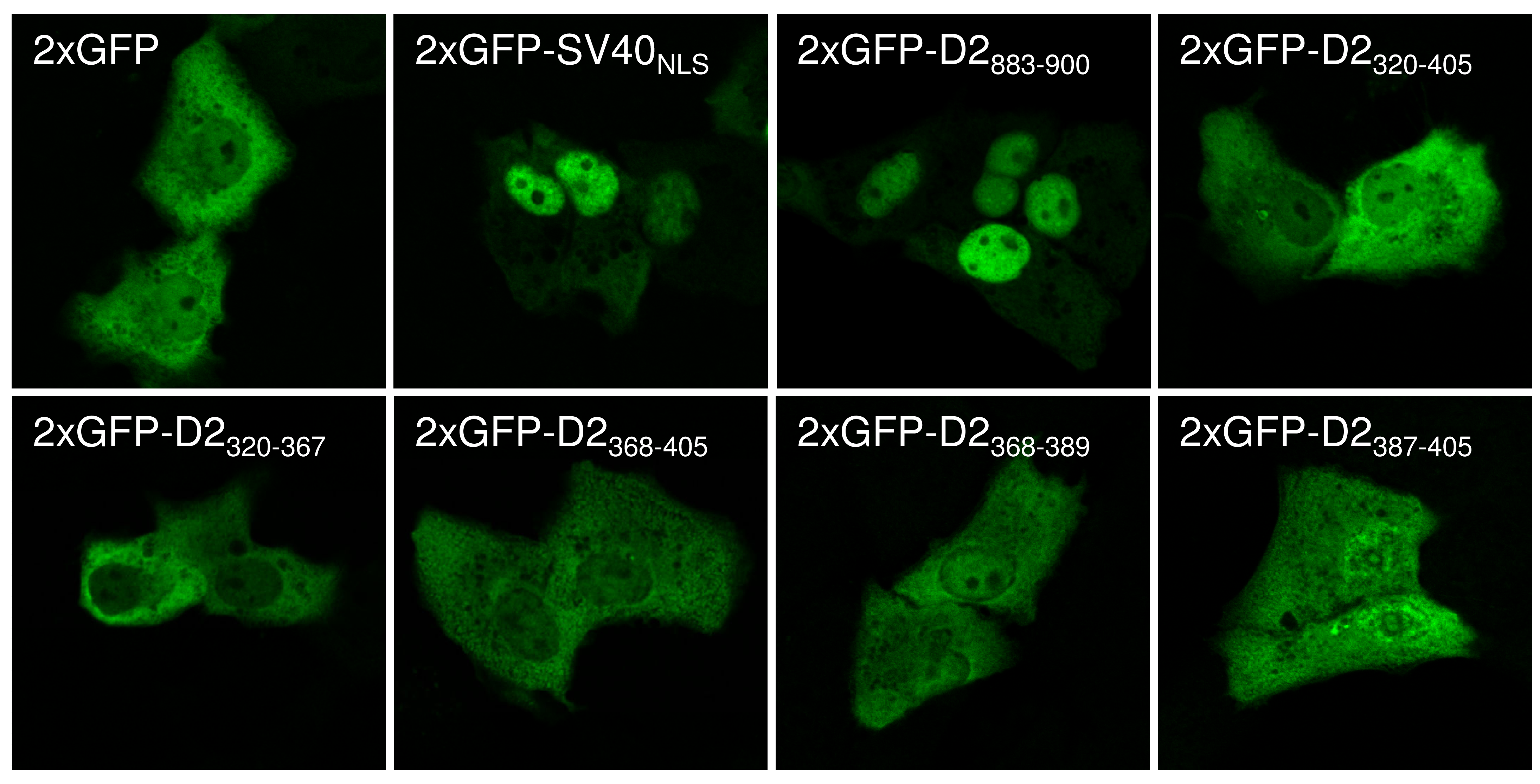

Supplement: S7 Fig — Vero cells were transfected with either 2×GFP or 2×GFP-fusion plasmids encoding residues 320–405, 320–367, 368–405, 368–389, 387 and 405 and 883–900 of DENV2 NS5, and fixed at 24h post-transfection. 2×GFP-fusion plasmid encoding SV40 NLS was included as a positive control for monopartite NLS. The cells were stained and images were captured as described previously. (TIF) [file ppat.1005886.s007.tif]

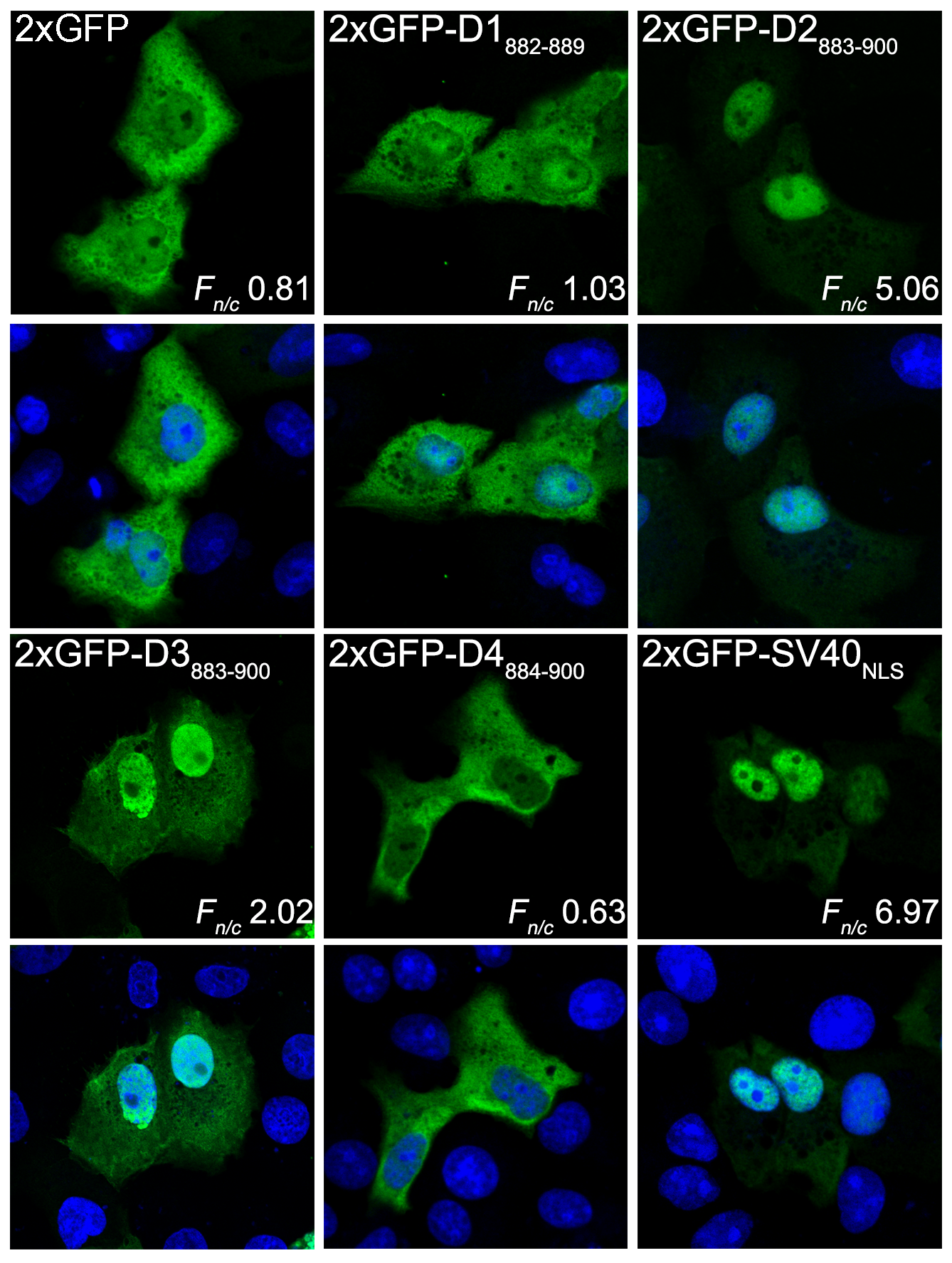

Supplement: S8 Fig — Vero cells were transfected with either 2×GFP or 2×GFP-fusion plasmids, and fixed at 24h post-transfection. 2×GFP-fusion plasmid encoding SV40 NLS was included as a positive control for monopartite NLS. Anti-GFP (ab6556 IgG, 1:1000) antibody was used for immunostaining and digitized images were captured by Zeiss LSM 710 upright confocal microscope by 40× oil immersion lens. Nuclear to cytoplasmic fluorescence ratio (F n/c) as previously described [28–30,42] are indicated and data are shown as mean F n/c, n ≥ 30 cells from a single assay, representative of two independent experiments. (TIF) [file ppat.1005886.s008.tif]

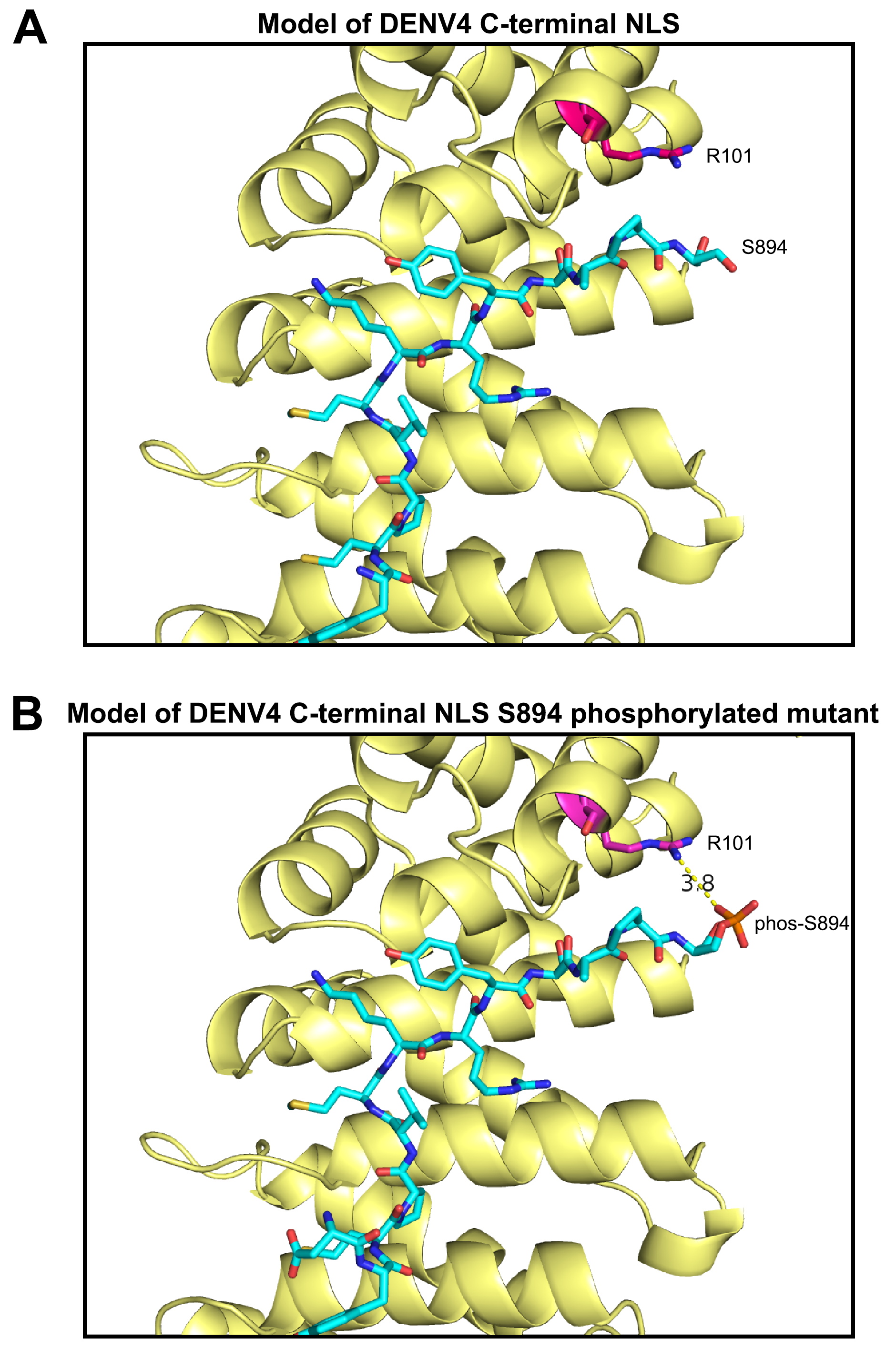

Supplement: S9 Fig — (A) Impα is shown as yellow ribbons, with key interacting R101 residue shown in magenta, the backbone of DENV4 C-terminal NLS model is shown in blue and (B) as in A, with the phosphorylated serine shown in orange. The models were made using COOT and the simple mutate function on the structure of DENV3 C-terminal NLS in complex with Impα. The serine was phosphorylated using “the phosphorylate this residue function” in the modelling tools of COOT. The distance was measured using the measure function in COOT and images were created using PyMOL. (TIF) [file ppat.1005886.s009.tif]

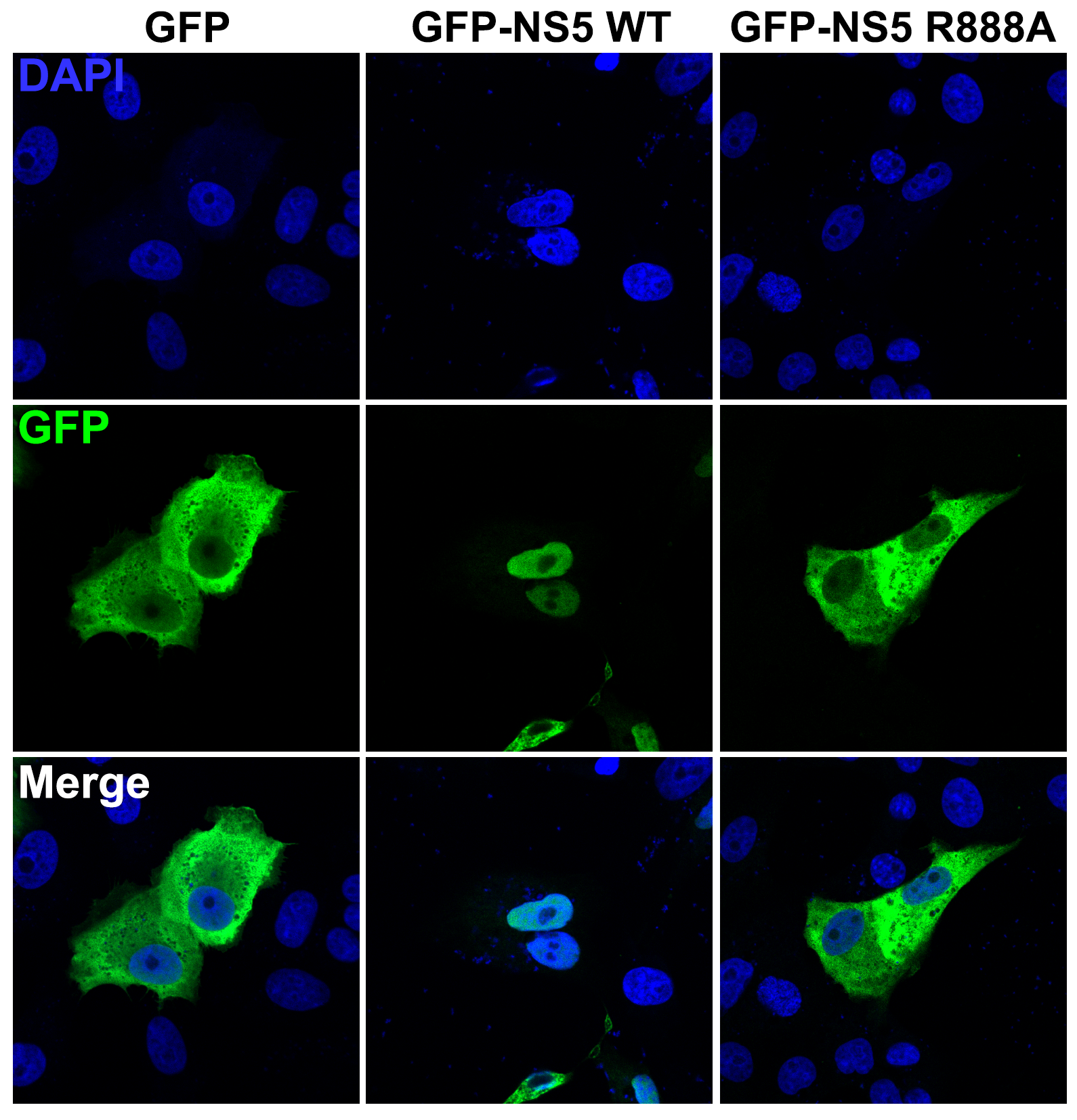

Supplement: S10 Fig — GFP, DENV2 GFP-NS5 WT and R888A protein constructs were transfected into Vero cells and fixed at 24 hr post-transfection. Anti-GFP (ab6556 IgG, 1:1000) antibody was used for immunostaining and digitized images were captured by Zeiss LSM 710 upright confocal microscope by 63× oil immersion lens. (TIF) [file ppat.1005886.s010.tif]

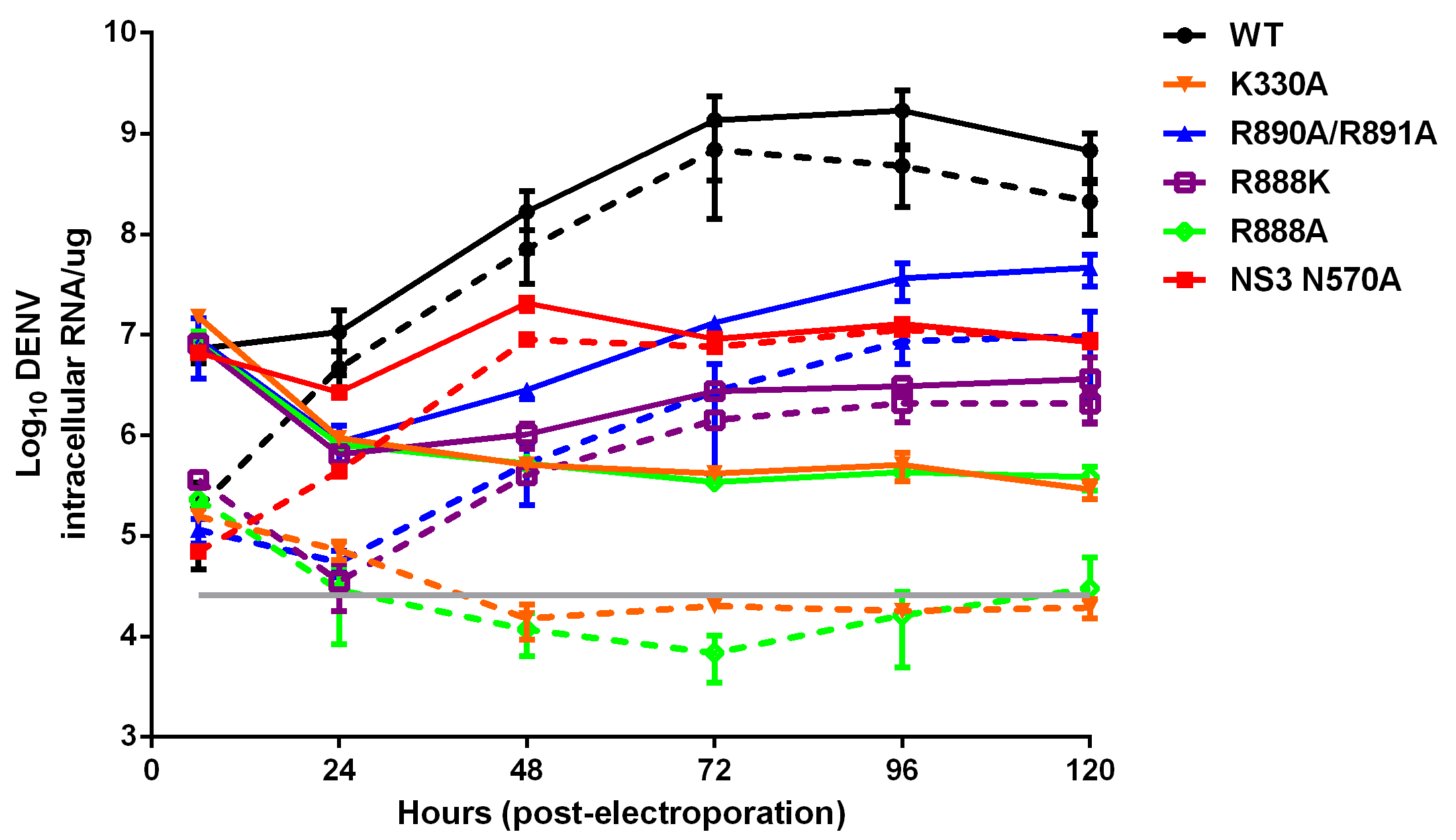

Supplement: S11 Fig — BHK-21 cells were electroporated with 10 μg of genomic-length RNA of WT DENV2 and mutants; supernatants and infected cells were harvested daily and consecutively for 5 days [59]. RNA was extracted from infected cells and absolute copy numbers of intracellular viral positive (solid line) and negative (dotted line) RNA was determined by real-time PCR. The values of viral genome numbers were normalized to actin expression level and absolute copy number of viral RNA per μg of RNA was plotted; data are shown as the mean ± SD from two dependent experiments. (TIF) [file ppat.1005886.s011.tif]
